# Tunable Bioresorbable Scaffolds With Marine Sulfated Polysaccharides for Small‐Caliber Vascular Grafts: A Multi‐Layered Strategy Combining Electrospinning and 4‐Axis Printing

**DOI:** 10.1002/adhm.202505314

**Published:** 2026-02-02

**Authors:** Gabriele Obino, Alberto Sensini, Tim ten Brink, Gabriele Nieddu, Tristan Bodet, Giovanni Andrea Deiana, Martijn van Griensven, Marilena Formato, Antonio J. Lepedda, Lorenzo Moroni

**Affiliations:** ^1^ Department of Biomedical Sciences University of Sassari Sassari Italy; ^2^ Department of Complex Tissue Regeneration MERLN Institute for Technology‐Inspired Regenerative Medicine Maastricht University Maastricht The Netherlands; ^3^ Department of Cell Biology‐Inspired Tissue Engineering MERLN Institute for Technology‐Inspired Regenerative Medicine Maastricht University Maastricht The Netherlands; ^4^ Department of Medicine Surgery and Pharmacy University of Sassari Sassari Italy

**Keywords:** small‐caliber tissue‐engineered vascular grafts, marine sulfated polysaccharides, electrospinning, 4‐Axis printing, endothelialization

## Abstract

The development of small‐caliber tissue‐engineered vascular grafts (sTEVGs) presents several challenges, including achieving balanced endothelialization, facilitating smooth muscle cell infiltration, preventing leakage, and ensuring anti‐thrombogenic properties, while maintaining mechanical strength sufficient to withstand physiological pressures, surgical handling, and suturing. Here, we present a multi‐layered polycaprolactone (PCL)‐based sTEVG using a combination of electrospinning and 4‐axis printing, providing precise control over scaffold porosity, fiber alignment, and tunable mechanical properties. To improve biocompatibility and hemocompatibility, the PCL nanofibers were functionalized with sulfated polysaccharides purified from the marine invertebrate *Holothuria tubulosa*, which significantly enhanced endothelialization and provided strong anti‐thrombogenic properties. The inner layer of tightly aligned electrospun nanofibers supported rapid formation of a mature endothelium, while preventing graft leakage even at supraphysiological pressure (>1100 mmHg). The middle layers, combining circumferential electrospun nanofibers and 4‐axis printed microfibers, increased scaffold porosity, and promoted adhesion, orientation and infiltration of human coronary artery smooth muscle cells (HCASMCs), facilitating functional tunica media formation. The outer layer of randomly oriented electrospun nanofibers contributed significantly to the mechanical properties of the graft, namely elasticity, toughness, burst pressure, and resistance to physiological vessel pressures, thus mimicking the tunica adventitia. The customizable four‐layered graft integrates structural and biological cues to address key limitations of sTEVGs, representing a valuableoff‐the‐shelf alternative to autologous grafts.

## Graphical abstract

A multilayered small‐caliber vascular scaffold combining electrospinning and 4‐axis printing is developed and biofunctionalized with marine sulfated polysaccharides from Holothuria tubulosa. The resulting construct exhibits enhanced hemocompatibility, tunable mechanical properties, and supports endothelial and smooth muscle cell adhesion and infiltration, highlighting its potential for next‐generation vascular grafts.

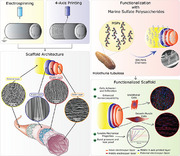

## 1 Introduction

Cardiovascular diseases (CVDs), including those related to small‐caliber vessels, such as Coronary Artery Disease (CAD) and Peripheral Artery Disease (PAD), are among the leading causes of morbidity and mortality worldwide [1, 2, 3]. Their prevalence continues to rise due to lifestyle factors and an aging global population [4, 5]. For many patients, conventional treatment options, such as coronary artery bypass grafting, rely heavily on the availability of suitable autologous vessels. However, in a significant number of cases, patients have vessels that are either unsuitable for grafting due to poor quality or are simply unavailable in sufficient quantities [6]. This lack of optimal grafting material drives the need for alternative solutions, such as Tissue‐Engineered Vascular Grafts (TEVGs), which have the potential to aid vascular surgery by providing an off‐the‐shelf alternative to autologous grafts [7, 8, 9].

While current TEVGs are effective for large‐caliber vessels with an internal diameter greater than 6 mm, their application to smaller‐caliber vessels, such as coronary arteries, has proven far more problematic. Small‐caliber vessels face unique challenges, particularly the high incidence of early graft occlusion. This occlusion is primarily due to poor endothelialization and adverse interactions between blood proteins and the scaffold material, which can lead to thrombosis [10, 11, 12, 13]. Therefore, the development of small‐caliber TEVGs (sTEVGs) must focus on promoting rapid endothelialization while preventing platelet activation and clot formation. Additionally, grafts need to support cell migration, proliferation, and tissue remodeling in situ to achieve long‐term functionality and integration with the native vasculature [14].

Despite advances in TEVG technology, several challenges remain, particularly for smaller vessels. Intimal hyperplasia, compliance mismatch, infection, and the long‐term adaptation of the graft to the host vessel environment, especially in pediatric patients, continue to present significant obstacles to clinical success [15, 16]. A promising approach to addressing these challenges involves the use of bioresorbable scaffolds, which mimic the native extracellular matrix (ECM) while guiding the formation of new tissue during the scaffold's degradation process. By carefully controlling the degradation rate, such scaffolds can provide temporary mechanical support until the host tissue has sufficiently remodeled and replaced the scaffolds’ biomaterial.

Among the various fabrication techniques explored for the development of sTEVGs, electrospinning is widely used because it allows for the fabrication of nanofiber scaffolds that closely mimic the size and orientation of ECM protein fibers. These nanofibers provide numerous sites for cell attachment, promoting cell colonization and tissue integration [14, 17, 18]. However, one of the key limitations of electrospun TEVG is the low porosity, due to the small fiber diameter. This low porosity impairs smooth muscle cell (SMC) infiltration and limits the deposition of ECM, which are critical for the long‐term stability and functionality of the graft [14, 19, 20]. Furthermore, electrospun scaffolds can struggle to maintain their tubular structure after bending, a factor that limits their utility in surgical applications where flexibility and durability are essential.

To overcome these limitations, we have developed a four‐layered polycaprolactone (PCL)‐based sTEVG using a combination of electrospinning and 4‐axis printing techniques. The first two layers of the scaffold are composed of electrospun nanofibers, with fibers aligned axially (First layer), facing the lumen of the tubular graft, or circumferentially (Second layer) to mimic the orientation of ECM in native vessels. The third layer consists of circumferential microfibers obtained by 4‐axis printing (4‐axis layer), which increases the porosity of the scaffold and facilitates SMC infiltration and ECM production. The final layer consists of randomly oriented electrospun nanofibers (External electrospun layer; EEL), which provide structural protection and prevent damage to the underlying layers. This multi‐layered design ensures that the scaffold has varying pore sizes across its structure, with smaller pores in the inner layers to prevent leakage and larger pores in the middle layers to promote cell migration and infiltration, while maintaining overall scaffold integrity.

Alongside its mechanical and structural optimizations, scaffold's hemocompatibility was improved by PCL nanofibers functionalization with sulfated polysaccharides from *Holothuria tubulosa*. These marine‐derived polysaccharides were chosen due to their known anticoagulant properties [21]. as well as their potential to improve hemocompatibility and endothelialization of nanofibrous PCL, as previously described [22].

In this study, we evidenced rapid endothelialization across the lumen surface of the scaffold, with human umbilical vein endothelial cells (HUVECs) forming a confluent monolayer within 3 days. Additionally, human coronary artery smooth muscle cells (HCASMCs) infiltrated the various strata of the middle layer while maintaining robust alpha smooth muscle actin expression, indicative of a contractile phenotype essential for optimal vascular wall function. Mechanical testing of the scaffolds demonstrated a non‐linear behavior, with tunable properties such as elastic modulus, yield stress, and burst pressure. This tunability is achieved through the multi‐layer design, where individual layers can be adjusted in thickness or removed as needed, enabling precise customization of the scaffold's mechanical characteristics to meet specific vascular requirements.

Overall, the multi‐layered, bioresorbable sTEVG, with its enhanced mechanical properties, anti‐thrombotic features, and superior endothelialization, might represent a valuable off‐the‐shelf, ECM customizable alternative to autologous grafts, with important implications on the quality of life of CVD patients requiring bypass surgery.

## 2 Results

### 2.1 Scaffold Design: Production and Fabrication

**FIGURE 1:**
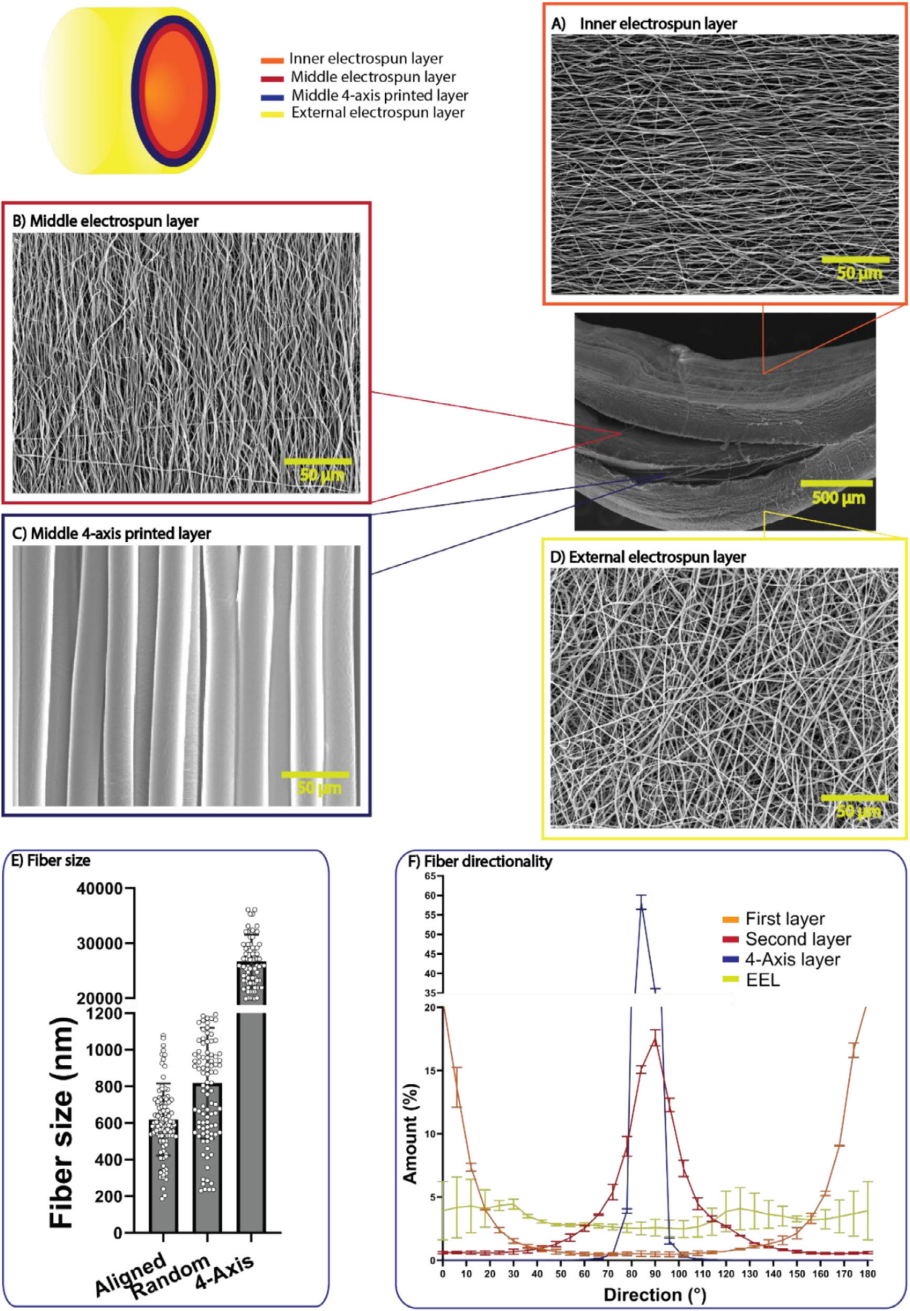
Scanning electron micrographs of the four distinct scaffold strata and a scheme of transverse section of the assembled graft. (A) Inner electrospun layer with nanofibers aligned parallel to the graft axis (×500; scale bar = 50 µm). (B) Second electrospun layer with nanofibers wrapped circumferentially (×500; scale bar = 50 µm). (C) 4‐axis–printed microfiber layer with microfibers circumferentially aligned (×500; scale bar = 50 µm). (D) Outermost electrospun layer with randomly oriented nanofibers, forming an adventitia‐like protective sheath (×500; scale bar = 50 µm). (E) Fiber size in the different layers. (F) Fiber directionality distribution of the different layers. Results expressed as mean ± SD (n = 5).

SEM images (Figure 1) confirmed the successful fabrication of a four‐layered PCL sTEVG with distinct micro‐ and nano‐architectures in each stratum. The inner layer (Figure 1A) comprised densely packed electrospun nanofibers aligned along the graft axis. Directly above this, a second electrospun layer (Figure 1B) featured nanofibers wrapped around the circumference of the graft, mimicking the native medial layer's fiber arrangement that resists vessel expansion under pressure, reproducing the hoop‐stress–bearing organization of the medial ECM. The third layer (Figure 1C) consisted of circumferentially deposited microfibers produced by a 4‐axis printing system, which created a more open, porous network designed to facilitate smooth muscle cell infiltration and de novo ECM deposition. Finally, an outermost electrospun layer of randomly oriented nanofibers (Figure 1D) enveloped the construct, providing a protective “adventitial” sheath that stabilizes the underlying structure and guards against delamination. Across all layers, fiber diameter and inter‐fiber spacing appeared uniform, and no defects or discontinuities were observed, demonstrating robust control over scaffold morphology and layer integration (Figure 1E,F).

### 2.2 Spectroscopic, Colorimetric, and Elemental Characterization of Scaffold Functionalization

The success of the scaffold functionalization was evaluated using a combination of complementary techniques and assays. ATR‐FTIR analysis revealed characteristic peaks at 3400 and 1650 cm^−^^1^, indicating effective crosslinking via EDC/NHS chemistry. Specifically, the broad peak at 3400 cm^−^^1^ corresponds to –OH and –NH stretching vibrations, while the peak at 1650 cm^−^^1^ corresponds to amide stretching. These peaks were observed only in the scaffolds functionalized with Ht and Hep (PCL‐Ht and PCL‐Hep), and were absent in untreated PCL scaffolds, confirming the chemical incorporation of marine sulfated polysaccharides onto the scaffold surfaces (Figure S1). In addition, Toluidine Blue staining, followed by quantitative analysis, was employed to assess the distribution and density of the functionalized polysaccharides across the scaffolds. The results showed a uniform distribution of the staining throughout the scaffold and revealed an average of 0.89 µg of sulfated polysaccharides per mg of scaffold for PCL‐Hep and 1.78 µg/mg for PCL‐Ht (Figure S2B,C).

The TBO assay was also used in our previous study to verify the functionalization stability over 1 month of incubation at 37°C in PBS and after storage for 12 months at room temperature in dry conditions. This demonstrated that the functionalization strategy and protocol exhibit excellent stability under both dry and physiological conditions [22].

This finding was further supported by EDX analysis, which provided semi‐quantitative insights into the surface elemental composition and confirmed the presence of sulfur‐containing groups derived from the polysaccharides. The analysis showed that sulfur accounted for an average of 0.045% of the total mass of the scaffolds in PCL‐Hep and 0.07% in PCL‐Ht (Figure S2A). Together, these methods provided robust and convergent evidence of the successful crosslinking of marine sulfated polysaccharides onto the PCL scaffolds.

### 2.3 Mechanical Characterization of Four‐Layer Bioresorbable Vascular Graft

**FIGURE 2:**
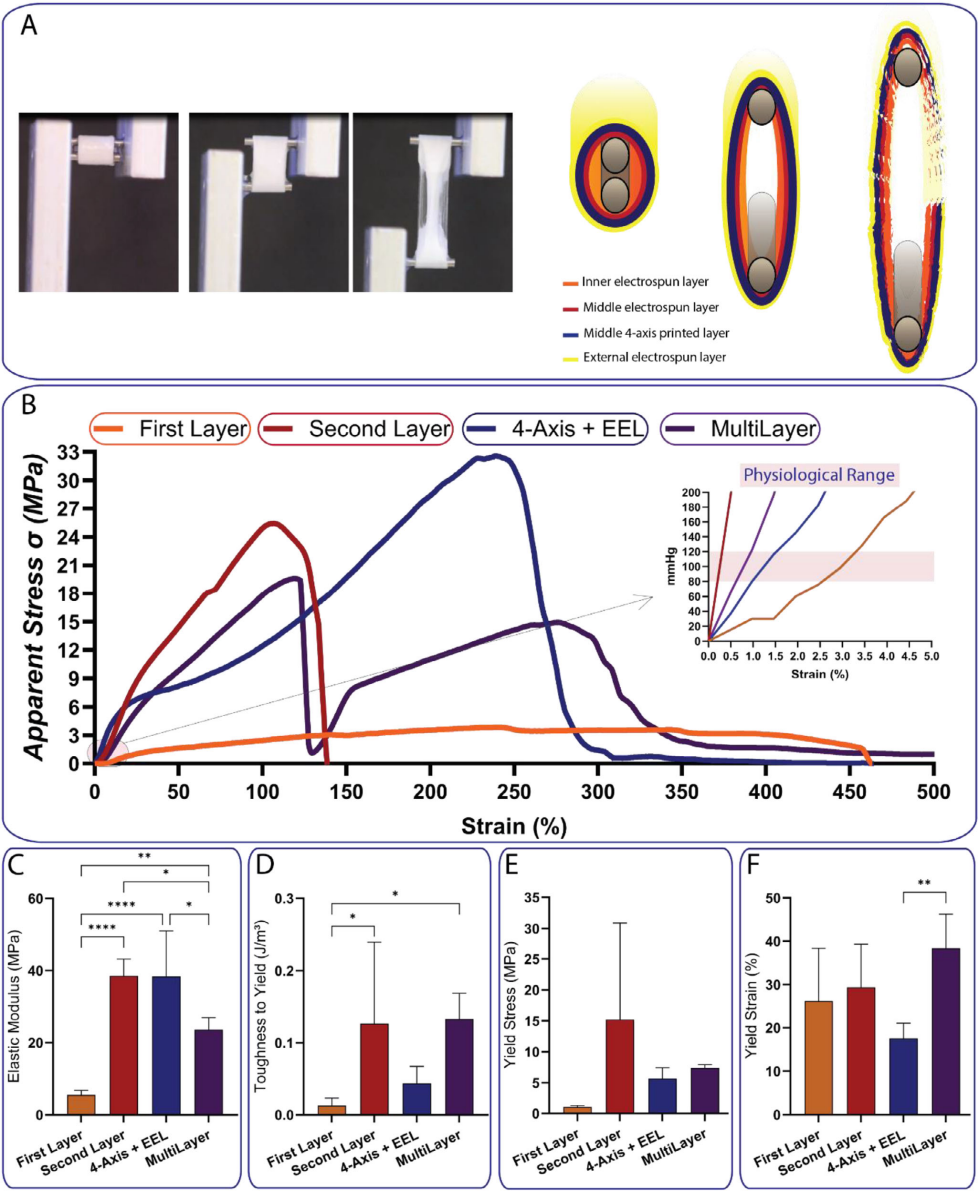
Mechanical characterization of multilayered TEVGs. (A) Tensile testing setup and schematic of the scaffold architecture. (B) Representative apparent stress‐strain curves for scaffolds with increasing numbers of layers. (C) Apparent elastic modulus, (D) apparent toughness to yield, (E) apparent yield stress and (F) yield strain for each scaffold configuration. Data: mean ± SD (n = 5). Statistics: ordinary one‐way ANOVA followed by Tukey's multiple comparisons test. *p‐value < 0.05; **p‐value < 0.01; ****p‐value < 0.0001.

**FIGURE 3:**
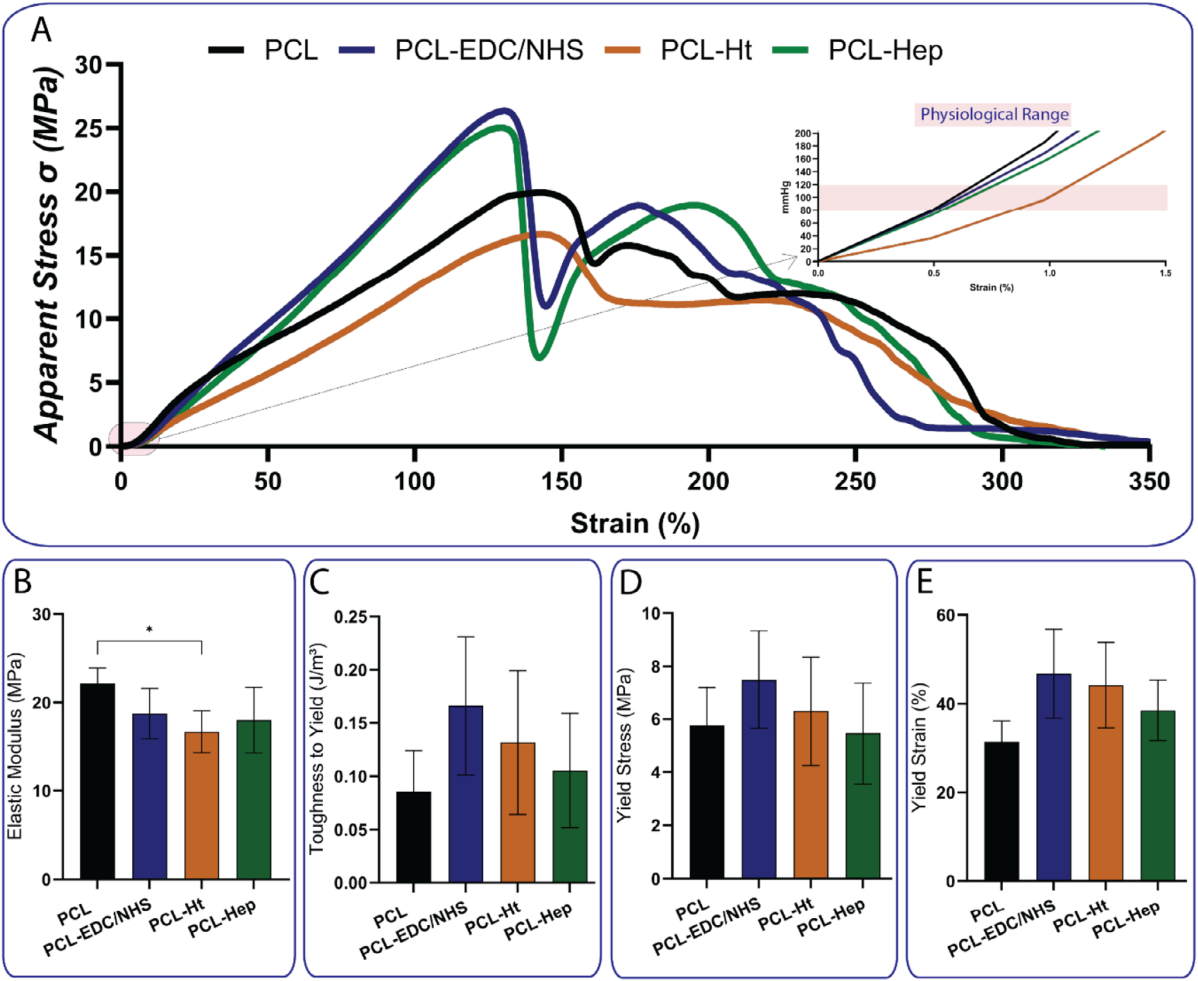
Mechanical properties of functionalized TEVGs. (A) Representative apparent stress–strain curves for PCL, PCL–EDC/NHS, PCL–Ht, and PCL–Hep scaffolds. (B) apparent elastic modulus, (C) apparent toughness to yield, (D) apparent yield stress and (E) yield strain for each scaffold functionalization. Data: mean ± SD (n = 5). Statistics: ordinary one‐way ANOVA followed by Tukey's multiple comparisons test. *p‐value < 0.05.

Uniaxial tensile testing of the electrospun and printed layers (V1) revealed a clear trade‐off between extensibility and strength. The innermost aligned nanofiber layer (First layer) was the most compliant, reaching very high elongation before failure, but it exhibited the lowest elastic modulus, toughness to yield and yield stress (Figure 2C–E) of all configurations. Successive addition of layers (circumferential nanofibers, printed microfibers, and an outer random nanofiber layer) progressively stiffened the construct (Figure 2C). In the stress–strain curves (Figure 2B), each extra layer shifted the curve upward: elastic modulus (Figure 2C) and toughness (Figure 2D) increased monotonically from the single‐layer scaffold to the full four‐layer graft. The four‐layered scaffold had the greatest toughness and highest elastic modulus. Importantly, the physiologically relevant zone of the stress‐strain curves is the pre‐yield one, defined by the yield force, yield stress, and yield strain. Beyond yield, the residual part of the curve mainly serves as a safety margin, delaying rupture if yield stress is exceeded. Thus, while the first layer provided high deformability with limited strength, the four‐layer assembly combined inner‐layer compliance with outer‐layer strength, resulting in superior energy absorption and structural reliability (Figure 2). The same observation can be appreciated by analyzing the net mechanical stress and elastic moduli of the different TEVG layers (Figure S3). Surface chemistry had only minor effects on bulk mechanics (Figure 3 and Figure S4). All PCL scaffolds retained a non‐linear ductile (non‐linear stress–strain) behavior (Figure 3A). Only PCL‐Ht displayed a slight reduction in elastic modulus (Figure 3B), which did not compromise overall mechanical integrity (Figure 3). Importantly, all functionalized scaffolds preserved high deformability comparable to unmodified PCL (Figure 3C–E).

**FIGURE 4:**
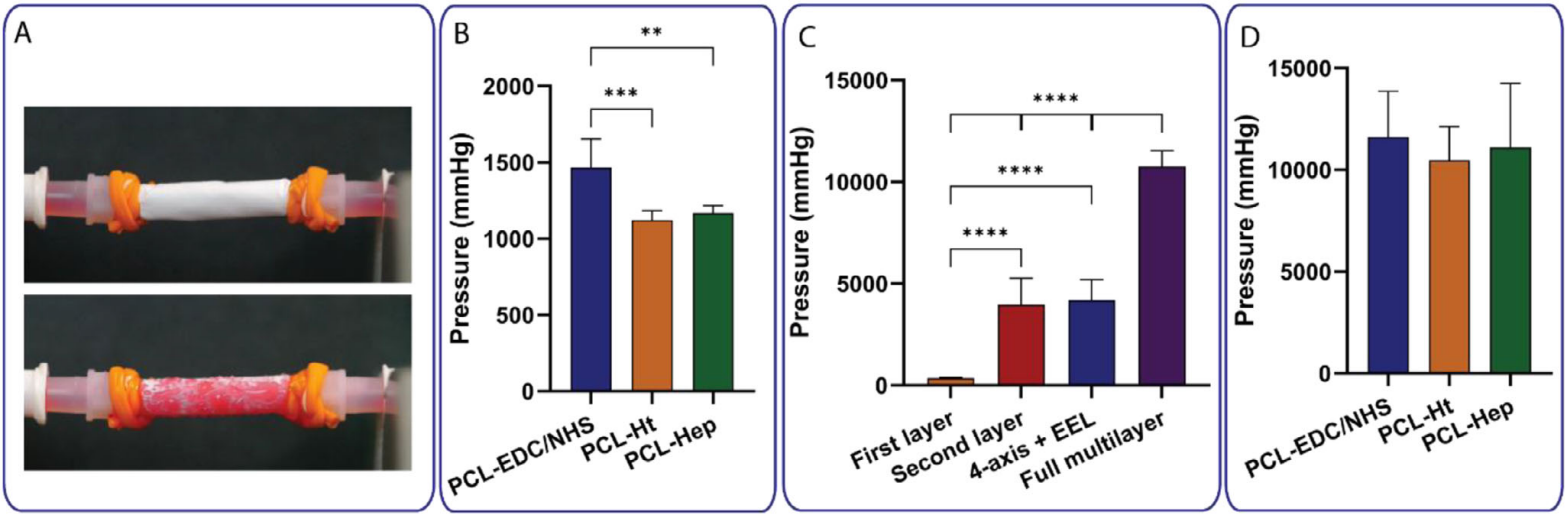
Burst pressure analysis of TEVGs. (A) Custom setup for direct burst pressure measurement. (B) Direct burst pressure values for 4‐layer TEVGs with different surface chemistries. (C) Theoretical burst pressure calculated for scaffolds with increasing layer numbers and for the 4‐layer construct. (D) Theoretical burst pressure for different functionalized scaffolds. Data: mean ± SD (n = 3). Statistics: ordinary one‐way ANOVA followed by Tukey's multiple comparisons test. **p‐value < 0.01; ***p‐value < 0.001; ****p‐value < 0.0001.

Burst pressure testing revealed remarkable improvements with scaffold layering (Figure 4). Direct burst experiments were conducted only on the fully assembled four‐layer tubular scaffolds using a customized platform (V2), showing that PCL–EDC/NHS grafts withstood ∼1467 ± 208 mmHg before rupture, while even the chemically modified variants, PCL–Ht and PCL–Hep, reached ∼1123 ± 68 mmHg and ∼1192 ± 81 mmHg, respectively (Figure 4B). All constructs exceeded 1000 mmHg, well above physiological systolic pressure, indicating strong in vivo pressure resistance. In contrast, indirect burst pressures for individual layers and intermediate assemblies were estimated using Laplace's law based on ring tensile data (Figure 4C,D). These calculations showed a clear layer‐dependent increase: the innermost nanofiber layer had the lowest theoretical burst (∼362 ± 31 mmHg), while the full four‐layer configuration reached a predicted burst of ∼10 759 ± 776 mmHg. This >25‐fold increase highlights how successive layering—particularly the incorporation of circumferential fibers, microfibers, and the outer sheath—contributed synergistically to wall tension capacity and hoop strength. Importantly, the direct burst results showed minimal difference between surface chemistries (PCL–EDC/NHS, PCL–Ht, PCL–Hep), all performing in the same supraphysiological range (>11 000 mmHg). This confirms that the structural architecture, not the surface modification, was the dominant determinant of burst pressure. In summary, the combination of theoretical and experimental results demonstrates that the multilayer design provides robust pressure resistance, far exceeding clinical benchmarks, and remains mechanically sound regardless of surface treatment (Figure 4).

Scaffold mechanics can be finely tuned by adding or removing individual layers (Figure S5). The incorporation of the compliant inner nanofiber layer markedly lowered the overall elastic modulus and increased extensibility, yielding a more elastic construct. Conversely, the addition of the circumferential nanofiber layer, and particularly the inclusion of two copies of that layer substantially raised both elastic modulus and yield strength, producing a more resistant scaffold (Figure S3). Compliance of the individual layers and of the multilayer TEVG, both before and after functionalization with sulfated polysaccharides, was calculated from the stress–strain curves (Figure S6). The inner nanofiber layer exhibited the highest compliance, followed by the multilayer TEVG (both functionalized and non‐functionalized). In contrast, the second electrospun layer and the 4‐axis + EEL configuration showed the lowest compliance and highest stiffness (Figure S6). By selectively adjusting layer number and order, it was possible to precisely balance scaffold elasticity, compliance, and mechanical strength.

### 2.4 HUVECs Culture on Vascular Scaffolds

**FIGURE 5:**
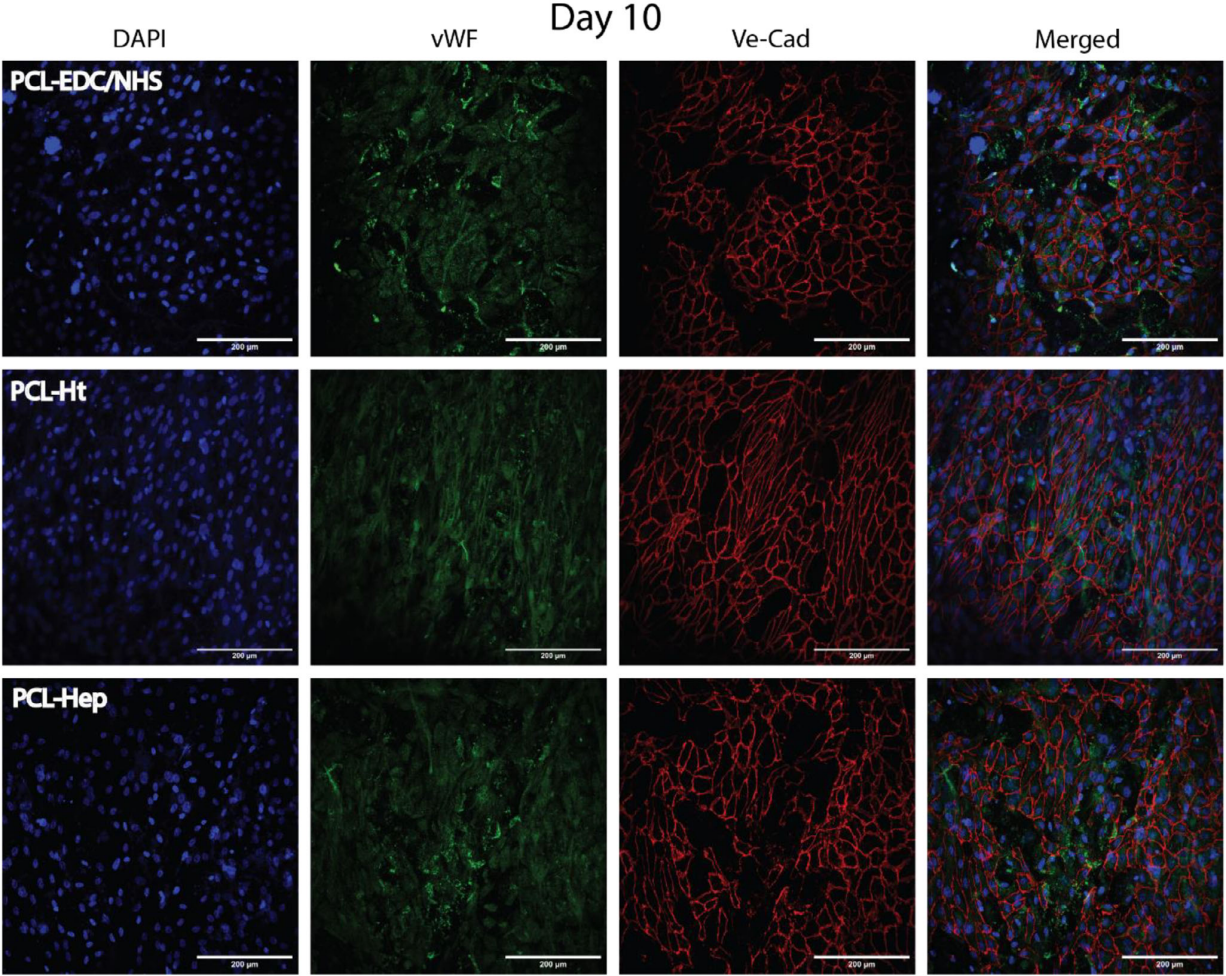
Immunofluorescent staining of HUVECs cultured under static conditions for 10 days on the luminal surface of TEVGs with different surface functionalizations: PCL‐EDC/NHS, PCL‐Ht, and PCL‐Hep. Nuclei are stained with DAPI (blue), cell‐cell junctions are labeled with VE‐Cadherin (red), and von Willebrand Factor (vWF) is shown in green. Images were acquired at 25× magnification. Scale bar: 200 µm.

**FIGURE 6:**
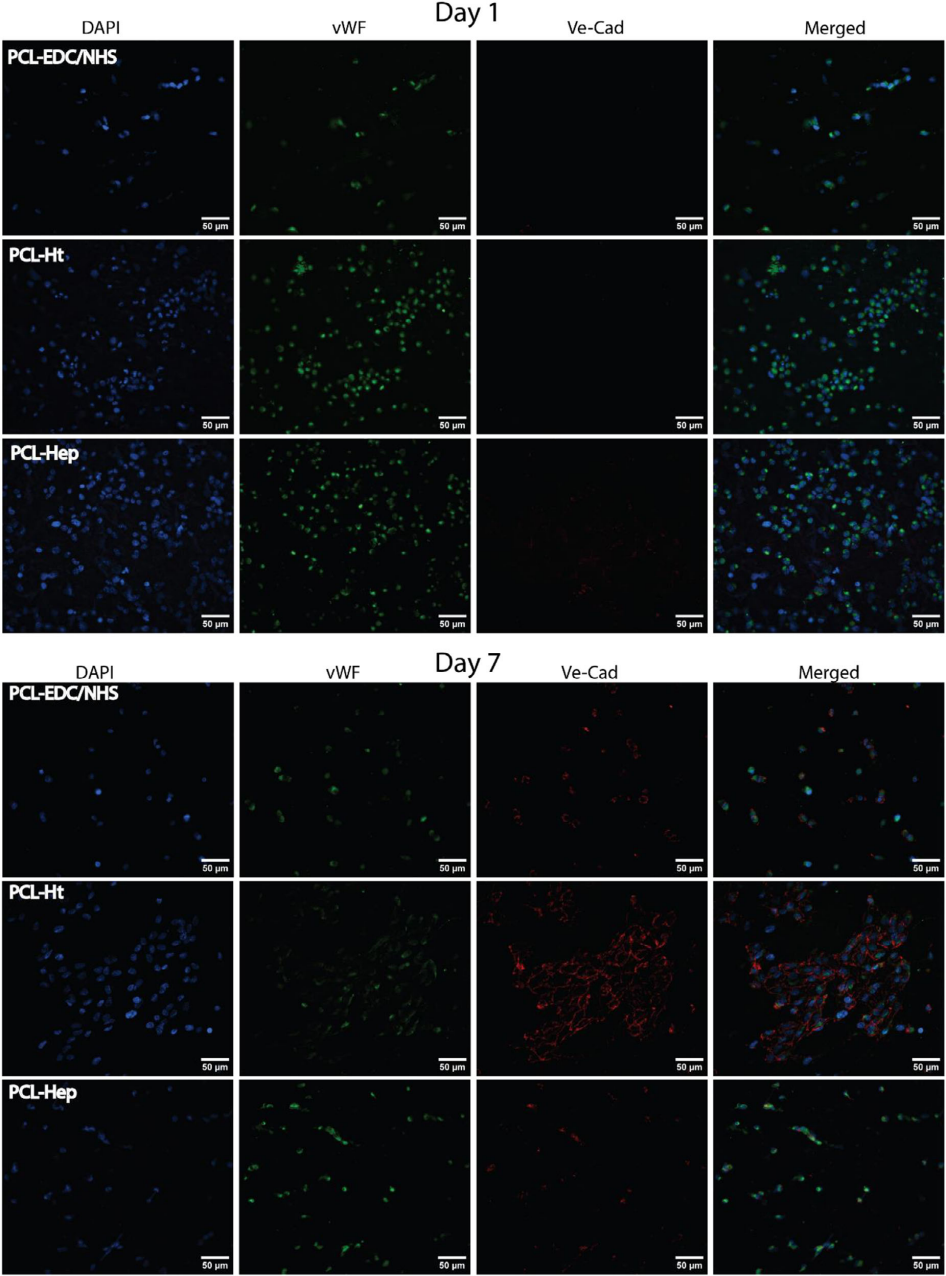
Immunofluorescent staining of HUVECs cultured under dynamic flow conditions on the luminal surface of TEVGs (PCL‐EDC/NHS, PCL‐Ht, and PCL‐Hep). Top panel represents cells at Day 1 post‐seeding; the bottom panel shows cells after 7 days of dynamic culture. Nuclei are stained with DAPI (blue), VE‐Cadherin marks cell‐cell junctions (red), and von Willebrand Factor (vWF) indicates endothelial phenotype (green). Images acquired at 40× magnification. Scale bar: 50 µm.

HUVECs were seeded onto the luminal surface of the scaffolds and cultivated for 21 days under static conditions (Figure 5) and 7 days under dynamic flow (Figure 6). Quantitative assessment via DNA content was performed at days 1, 10, and 21 for statically cultured constructs (Figure S7), complemented by comprehensive immunofluorescence analysis of the tissue‐engineered vascular graft (TEVG) surface.

#### 2.4.1 Static Culture Outcomes

Under static conditions, HUVECs demonstrated differential colonization patterns depending on scaffold surface modification. Ht‐treated scaffolds (PCL‐Ht) supported significantly more rapid and extensive endothelial coverage compared to both PCL‐EDC/NHS controls and PCL‐Hep. By day 21, endothelial cells on Ht‐treated scaffolds exhibited characteristics of a mature, functional endothelium characterized by a confluent monolayer without intercellular gaps and pronounced cellular alignment along the nanofiber orientation axis (Figure 5).

#### 2.4.2 Dynamic Flow Culture Results

In the dynamic flow system setup (Figure S8), initial cellular attachment (6 h post‐static seeding) revealed incomplete coverage across all scaffold types (Figure 6 top panel). Notably, both PCL‐Ht and PCL‐Hep scaffolds demonstrated substantially higher initial cell adhesion compared to unmodified PCL constructs. Immunocytochemical analysis at this early timepoint showed positive expression of von Willebrand factor (vWF) with clear nuclear staining, while VE‐cadherin, a critical adherens junction protein essential for endothelial barrier function, remained undetectable, confirming the immature state of the nascent endothelium.

Following 7 days of culture under physiological flow conditions, endothelial cells across all scaffold types began expressing VE‐cadherin at cell‐cell interfaces, indicating the initiation of adherens junction formation. This expression pattern is consistent with the known role of VE‐cadherin in establishing intercellular connections during endothelial maturation. Significantly, only Ht‐treated scaffolds supported the development of a near‐confluent endothelial monolayer, with cells exhibiting fully formed adherens junctions and adopting the characteristic hexagonal cobblestone morphology associated with functional endothelium (Figure 6 bottom panel).

### 2.5 CASMC Attachment, Proliferation, and Morphological Response to Scaffold Chemistry

**FIGURE 7:**
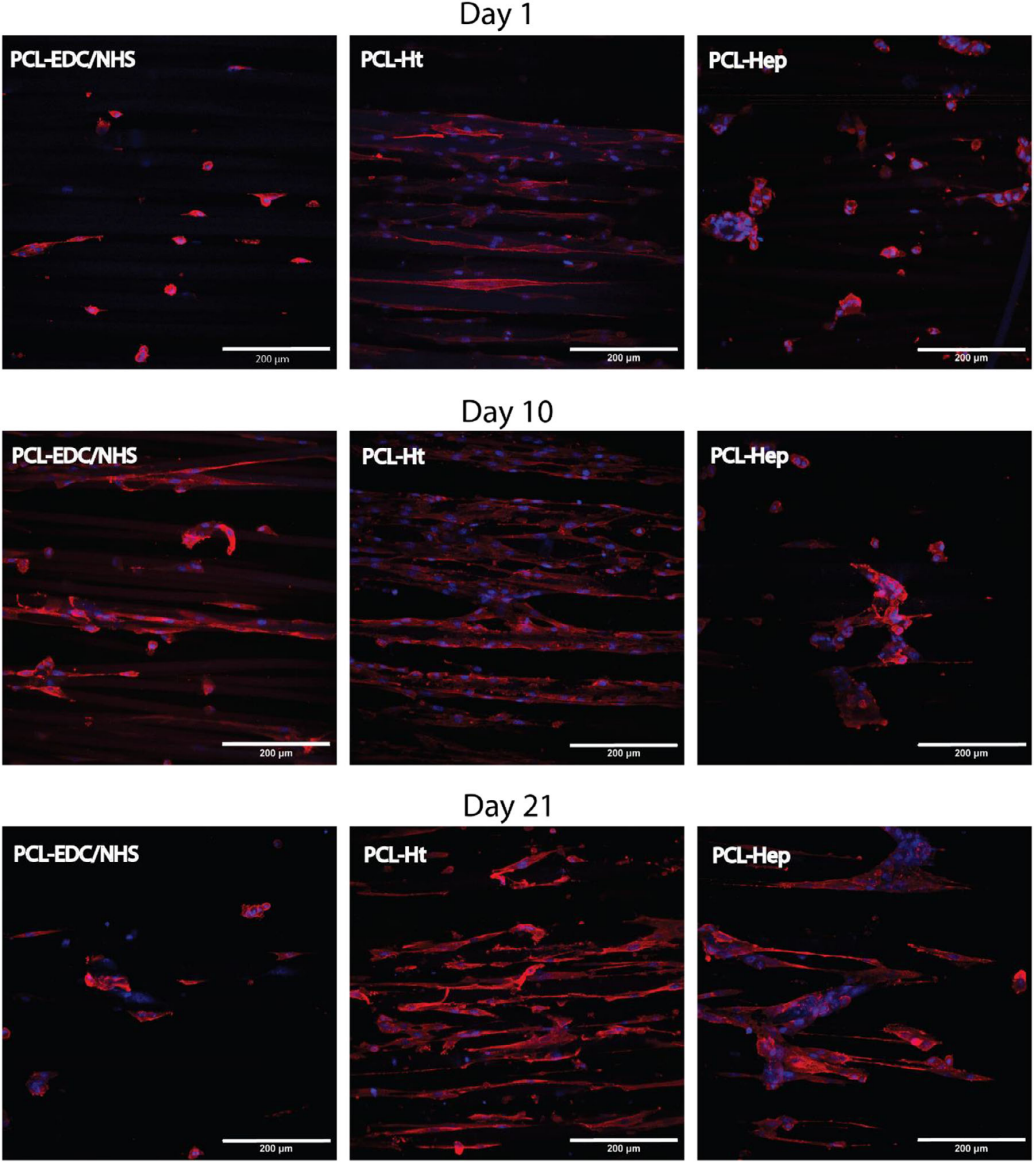
Immunofluorescent staining of CASMCs cultured under static conditions on the external surface of functionalized tubular scaffolds (PCL‐EDC/NHS, PCL‐Ht, and PCL‐Hep). CASMCs were seeded on the microfiber outer layer of scaffolds. Red fluorescence indicates α‐smooth muscle actin (α‐SMA), while blue fluorescence (DAPI) marks nuclei. Images acquired at 25× magnification. Scale bar: 200 µm.

CASMCs were seeded onto the microfiber outer layer of each scaffold and cultured under static conditions for 21 days (Figure 7 and S9). Confocal microscopy z‐stack analysis demonstrated robust cell infiltration across all scaffold types (Figure 7 and V3,V4 and V5). Quantitative DNA assay results revealed comparable cell attachment and proliferation among PCL‐EDC/NHS, PCL‐Ht, and PCL‐Hep scaffolds at early timepoints (days 1 and 10), with no statistically significant differences observed (Figure S9). However, by day 21, both PCL‐Ht and PCL‐EDC/NHS scaffolds maintained significantly higher cell populations compared to PCL‐Hep scaffolds (p < 0.001), indicating a potential inhibitory effect of heparin on long‐term CASMC maintenance. Longitudinal analysis of DNA content across timepoints revealed relatively stable cell populations on PCL‐Ht and PCL‐EDC/NHS scaffolds throughout the culture period, while PCL‐Hep scaffolds exhibited a significant decrease in cell number by day 21.

α‐SMA immunofluorescence analysis revealed striking phenotypic differences dependent on scaffold chemistry. CASMCs cultured on PCL‐Ht scaffolds exhibited the characteristic contractile phenotype, with elongated, spindle‐shaped morphology aligned parallel to fiber orientation. These cells displayed well‐defined α‐SMA‐positive stress fibers and developed multilayered structures resembling the native arterial tunica media architecture (Figure 7). In contrast, CASMCs on PCL‐EDC/NHS surfaces predominantly adopted rounded or partially spread morphologies, forming small, loosely associated clusters with intermittent and diffuse α‐SMA expression (Figure 7, middle panel). The most pronounced phenotypic deviation occurred on PCL‐Hep scaffolds, where CASMCs assembled into dense spheroidal aggregates with minimal cellular elongation and disrupted α‐SMA fiber organization, maintaining similar overall cell numbers until day 10 (Figure 7, bottom panel). Collectively, these findings demonstrate that while scaffold chemistry minimally influences initial CASMCs attachment and early proliferation, it substantially impacts long‐term cell retention and phenotypic maintenance, with PCL‐Ht scaffolds best supporting the contractile CASMCs phenotype critical for vascular tissue engineering applications.

## 3 Discussion

### 3.1 Biomimetic Scaffold Architecture

The extracellular matrix (ECM) of vascular arteries is characterized by a complex, multilayered architecture and a distinct cellular composition, with each layer contributing uniquely to the overall vascular function [23, 24]. The intima, media, and adventitia each plays a crucial role in maintaining vessel integrity: the intima minimizes thrombosis, the media, particularly in elastic arteries, dampens the pulsatile pressure generated by cardiac output, and in the muscular arteries, such as the coronary vessels, dynamically regulate blood flow to meet the metabolic demands of the myocardium. This intricately orchestrated structure is essential for preventing clot formation, inhibiting leakage, and ensuring the effective transmission and modulation of hemodynamic forces throughout the vascular network. Numerous researchers have sought to engineer vascular grafts that replicate the complex architecture, fiber size, and orientation of native blood vessels [25]. Electrospinning provides a versatile method to fabricate nanofibers with dimensions and alignment similar to ECM proteins, enhancing surface area and cellular adhesion. However, electrospun scaffolds alone often exhibit limited pore size, which restricts effective cell infiltration and remodeling [18]. Conversely, advanced techniques like melt electrowriting and 3D printing can create open, porous scaffolds that improve cellular infiltration [26, 27, 28, 29, 30]. Yet, the associated larger pore sizes may compromise barrier function, increasing the risk of leakage.

Our approach addresses these limitations by combining electrospinning and 4‐axis printing to create a multilayer scaffold that mimics the layered structure of native vessels. The innermost layer of aligned electrospun nanofibers offers a dense, compliant, and adhesive substrate that supports endothelial alignment and monolayer formation. The adjacent circumferential electrospun layer adds tunable stiffness and toughness. Centrally, a printed microfiber layer introduces a porous architecture that enables SMC infiltration and circumferential alignment on different strata, resembling the tunica media. Finally, an outer layer of randomly oriented nanofibers simulates the adventitia, reinforcing the scaffold and preventing delamination.

Effective sTEVG development faces significant challenges due to thrombogenic properties and poor endothelialization of the synthetic scaffold. To enhance endothelialization and reduce leakage, many researchers have applied protein coatings such as fibrin or collagen/gelatin. However, these coatings are subject to degradation over time, leading to cell detachment, which may trigger platelet activation and thrombosis [31, 32, 33, 34].

Building on our previous work, we leveraged the well‐established anticoagulant properties of fucosylated chondroitin sulfate isolated from the echinoderm *Holothuria tubulosa* [21]. to improve the hemocompatibility and endothelialization of nanofibrous PCL scaffolds, while simultaneously reducing platelet adhesion and aggregation [22]. In this study, the previously established approach was applied to engineer an innovative graft that combines the superior mechanical properties of the fourlayer bioresorbable PCL scaffold with the biological properties of these intriguing polysaccharides [22]. Interestingly, as they are covalently immobilized on the scaffold surface via EDC/NHS chemistry, their biological effects arise from stable surface presentation rather than sustained release.

### 3.2 Mechanical Properties

The four‐layer PCL scaffold exhibits mechanical properties and architecture tailored to mimic native vessels. Its burst pressure and tensile strength match or exceed those of autologous grafts. The soft, aligned nanofiber inner layer provides low‐strain compliance and a non‐linear stress–strain response similar to arteries [35], while the circumferential nanofiber, printed microfiber, and outer random‐fiber layers reinforce hoop strength and prevent delamination.

Importantly, the circumferential layer produced by 4‐axis printing contributes not only to mechanical reinforcement but also to interlayer integration: during deposition, molten PCL microfilaments partially fuse with the underlying electrospun nanofibrous layer, promoting mechanical continuity without disrupting nanofiber morphology. Subsequently, the outer randomly oriented electrospun layer is deposited directly onto the assembled structure, enabling fiber integration with the printed layer and further unifying the scaffold. In addition, progressive cellular infiltration and ECM deposition within the graft are expected to enhance interlayer cohesion over time, potentially reducing the risk of long‐term delamination following implantation. This layered strategy effectively decouples graft strength from compliance, avoiding the maladaptive stiffness of PET/ePTFE [36].

Burst pressure was assessed using two complementary approaches: a direct fluidic test, and an indirect estimation derived from tensile force at failure using a modified form of Laplace's law. The direct method involved real‐time internal pressurization of the scaffolds within a closed‐loop system, capturing the influence of geometric deformation, potential leakage, and material imperfections. In contrast, the indirect method offered a theoretical estimation using an idealized model, assuming a perfectly cylindrical geometry with thin walls, to convert tensile force into theoretical burst pressure. Measured burst pressures ranged from 1100 to 1700 mmHg, with indirect calculations reaching over 10 000 mmHg. These values compare favorably with native vessel benchmarks—for example, saphenous veins (1600–2400 mmHg) and internal mammary arteries (3200–4225 mmHg) [11, 37, 38]. and are consistent with earlier‐generation TEVGs, which typically targeted burst pressures of 1500–3500 mmHg [37, 39]. Importantly, all fully assembled scaffolds, regardless of surface functionalization, exhibited equivalent high‐level burst performance, confirming that mechanical integrity is predominantly governed by scaffold architecture rather than surface chemistry [40].

Reported tensile strengths for native vessels vary widely due to methodological differences, with moduli ranging from ∼1.5 MPa to >40 MPa [41, 42, 43, 44]. Such variability arises from differing approaches to modulus calculation. Some studies define elastic modulus as the steepest linear slope prior to failure, emphasizing structural strength, while others focus on the initial, low‐strain region of the curve, reflecting physiological compliance. For instance, Yokoyama et al. used the linear portion of the stress–strain curve dominated by elastin (strain 0–0.6) to define an “elastin‐associated” modulus, which closely matched native compliance in the physiological strain range [45]. Similarly, Soletti et al. explicitly defined elastic modulus as the slope of the stress–strain curve within physiological arterial pressure (∼80–120 mmHg), emphasizing the importance of evaluating compliance in the actual functional range [44]. In contrast, Bouchet et al. analyzed bilinear ePTFE behavior and selected the higher modulus (∼17.4 MPa), derived from the final linear region, to represent mechanical strength under near‐failure conditions [46]. Such methodological differences highlight why reported elastic modulus values should be interpreted with caution [18]. In line with studies that emphasize structural strength, we calculated the elastic modulus as the slope of the linear segment of the stress–strain curve before yield. Our scaffold, while stiffer than some native vessels, retains ductile, non‐linear behavior due to its layered design. For comparison, Han et al. fabricated a three‐layer electrospun graft (inner PLCL/gelatin, middle PLGA, outer PCL) whose tensile strength (5.2 ± 0.7 MPa) and modulus (35.9 ± 7.7 MPa) greatly exceeded that of a porcine coronary artery (2.6 and 1.0 MPa, respectively) [47, 48]. Likewise, Wu et al. reported radial strength ∼5.1 MPa, matching the human aorta and exceeding coronary values [47]. Our multilayered structure delivers comparable performance to what is reported in the literature while preserving inner‐layer compliance, enabling graded stiffness and mechanical anisotropy as in native arteries [47, 49, 50]. To assess physiological performance, we separately derived compliance from the stress–strain curve in the approximate arterial pressure range (∼80–120 mmHg). The inner electrospun layer exhibited high compliance, mimicking the elastic behavior of native elastin fibers, while the outer layers showed reduced compliance, reflecting the load‐bearing role of collagen in native vessel walls. However, translating tensile data into physiologically relevant compliance entails limitations [51]. The stress–strain approach inherently assumes idealized vessel geometry and material properties: a long, cylindrical, thin‐walled, homogeneous, and incompressible tube. In reality, native vessels exhibit taper, branches, residual stresses, and complex multi‐layered architecture, all of which affect local mechanical responses [51, 52]. Thus, while our stress–strain‐based compliance analysis offers a useful approximation, it should be interpreted in light of these geometric and biomechanical assumptions.

Relative to conventional synthetic grafts, the multilayer design offers distinct mechanical advantages. Standard materials like Dacron (PET) and ePTFE are very stiff [36, 53] and virtually noncompliant, which causes a compliance mismatch at anastomoses [54]. This mismatch is implicated in intimal hyperplasia and graft failure [54]. Our bioresorbable scaffold, in contrast, offers a native‐like mechanical profile. The elastic inner layer allows axial and circumferential stretch, while outer layers resist radial deformation. This layered design mimics the functional anisotropy of native arteries and may reduce intimal hyperplasia and graft failure.

In the context of TEVG literature, these results are encouraging. Earlier work has shown that layering can decouple compliance and strength: for example, adding helical or circumferential reinforcement to a fibrous scaffold dramatically boosts burst pressure without compromising endothelial‐friendly compliance as demonstrated by our scaffolds [47]. The polysaccharide surface coatings (Ht, Hep) modestly weakened individual fibers but did not prevent the full four‐layer graft from achieving native‐level bursts. In fact, even the weakest functionalized graft exceeded 1000 mmHg by a comfortable margin. Importantly, all graft variants after full assembly performed similarly in burst, indicating that any minor effects of polysaccharide functionalization are overcome by the structural architecture.

Finally, because the graft is bioresorbable, mechanical integrity will evolve as tissue forms. Ideally, polymer strength should degrade in concert with new extracellular matrix deposition to avoid aneurysm [55]. Our high initial stiffness ensures safety at implantation, and the layered architecture may allow gradual load transfer: as the inner PCL nanofibers soften or degrade, SMC‐deposited collagen in the media can take up stress.

A key advantage of our multilayer scaffold is its finely tunable mechanical profile, achieved through strategic adjustment of layer composition and architecture. The supplementary analysis demonstrates that scaffold elastic modulus, toughness, and tear resistance can be precisely modulated by adding or omitting specific structural layers. For instance, incorporating the most elastic inner nanofiber layer reduces elastic modulus while enhancing extensibility and toughness, offering compliance closer to that of native vessels. In contrast, adding or doubling the circumferentially aligned nanofiber layer substantially increases the elastic modulus, yield strength, and tear resistance. Such mechanical tunability is especially important for applications like coronary artery bypass grafting, where grafts must endure continuous cyclic strain, dynamic curvature, and localized compressive forces imposed by myocardial motion. The modularity of the scaffold also supports patient‐ and site‐specific customization: its layered configuration can be adapted to match the mechanics of different vascular beds, from high‐flow arteries to more compliant veins. This adaptability enhances the translational potential of the platform, allowing graft performance to be tailored to specific clinical contexts and anatomical demands.

### 3.3 Endothelialization and Hemocompatibility

Rapid endothelialization is essential for long‐term graft patency and thromboresistance. Among the scaffold variants, those functionalized with *Holothuria tubulosa*‐derived sulfated polysaccharides (PCL‐Ht) demonstrated the most favorable endothelial response. Under static HUVEC culture conditions, PCL‐Ht scaffolds promoted the formation of a functional endothelium, outperforming both PCL‐Hep and PCL‐EDC/NHS controls. Within a few days of culture, endothelial cells established confluent, uniformly aligned monolayers with evident cell‐cell interactions, indicative of advanced structural organization and endothelial maturation.

Under physiological flow, only PCL‐Ht scaffolds developed near‐confluent cobblestone endothelium by day 7, with well‐defined VE‐cadherin junctions. These findings align with our previous study [22]. The enhanced endothelial response likely stems from two factors. First, the Ht polysaccharides increase scaffold hydrophilicity and present bioactive sulfate motifs that support cell adhesion and anti‐thrombosis properties, as seen in other studies of marine sulfated polysaccharides [22]. Second, the aligned nanofibrous topography itself exerts a strong contact‐guidance effect: endothelial cells preferentially adhere to and elongate along aligned fibers, which accelerates monolayer formation [56]. In summary, the combination of the inner aligned layer and the Ht functionalization promoted an early, functional and anti‐thrombogenic endothelial barrier, a critical first step to prevent thrombosis and maintain graft patency.

### 3.4 Smooth Muscle Cell Infiltration and Phenotype

While endothelial integrity is essential for luminal patency, medial integration by contractile smooth muscle cells (SMCs) is equally critical to long‐term vascular function. The mid‐layer, fabricated using 4‐axis printing, supported extensive SMC infiltration across all scaffold variants, reflecting the design's high porosity. Early after seeding (days 1–10), CASMC attachment and proliferation were comparable on all scaffolds. However, by day 21 we observed stark differences driven by surface chemistry. Heparin‐functionalized grafts (PCL‐Hep) exhibited a pronounced drop in CASMC numbers (p<0.001), whereas PCL‐Ht and non‐coated scaffolds maintained high cell counts. Heparin is a known vascular SMC mitosis inhibitor—its antiproliferative effect has been documented for decades [57]—and our data suggest that the immobilized heparin on the graft surface impaired long‐term CASMC survival or proliferation. Moreover, the physical phenotype was remarkably altered: on PCL‐Hep, CASMCs formed dense, rounded spheroids with minimal α–smooth muscle actin organization. By contrast, CASMCs on PCL‐Ht remained well‐spread, elongated and aligned to the fibers, with abundant α‐SMA stress fibers and multilayered structures resembling the native tunica media.

The heparin‐associated phenotype is consistent with the literature: surface‐bound heparan sulfates (and heparin) render the substrate anti‐adhesive to SMCs, disrupting focal adhesions and preventing normal cytoskeletal tension [58]. Lundmark et al. demonstrated that smooth muscle cells on fibronectin coated with heparin lose focal contacts and stress fibers [58]—mirroring our observations on PCL‐Hep. In contrast, PCL‐Ht scaffolds appear to preserve the contractile phenotype of the CASMCs limiting excess proliferation. PCL‐Ht grafts uniquely support both rapid endothelial sealing and stable CASMC colonization in a contractile state—two key requirements for long‐term graft integration.

### 3.5 Future Perspectives and Clinical Implications

This multilayer, bioresorbable scaffold represents a compelling platform for next‐generation small‐caliber vascular grafts. Its modular architecture offers the flexibility to tailor mechanical and biological properties to specific clinical contexts. For example, increasing the inner‐layer thickness while reducing circumferential reinforcement may yield more compliant grafts suitable for low‐pressure venous systems, whereas augmenting the circumferential fiber content could enhance radial strength for high‐pressure muscular arteries. Compared to traditional electrospun PCL grafts—which suffer from low compliance, slow degradation, poor cell infiltration, and mid‐term calcification [59, 60]—our layered architecture mitigates these limitations by distributing mechanical loads and enhancing biofunctionality. Notably, long‐term studies suggest that persistent PCL strands may trigger neointimal calcification, likely due to osteochondrogenic transformation of smooth muscle cells under chronic mechanical stress [61, 62]. In contrast, the effective cell infiltration observed in our study may mitigate this pathological response.

Future optimization could involve polymer blends with distinct degradation kinetics or mechanical properties, as well as layer‐specific functionalization to independently modulate endothelial and smooth muscle behavior.

Furthermore, in vivo studies in relevant animal models are mandatory to evaluate native ECM deposition, scaffold resorption, and long‐term integration under physiological conditions. Ultimately, the synergistic combination of rapid endothelialization, stable smooth muscle integration, and tunable mechanical behavior positions this scaffold as a promising candidate for clinical translation. With further development, particularly in terms of bioreactor‐based preconditioning and scalable fabrication, this platform may enable the production of patient‐specific, off‐the‐shelf vascular grafts that overcome the key limitations of current synthetic options.

## 4 Conclusion

We developed and validated a modular, multilayered bioresorbable vascular graft that integrates tailored mechanical performance with endothelial and smooth muscle cell compatibility. By strategically combining electrospun and four‐axis printed layers, we achieved a scaffold with tunable stiffness, high burst resistance, and structural integrity suitable for arterial applications. Surface functionalization with marine‐derived polysaccharides supported endothelialization and hemocompatibility without compromising mechanical properties. The graft's design flexibility enables adaptation to site‐specific vascular demands, making it a promising platform for next‐generation tissue‐engineered vascular replacements.

## 5 Experimental Section

The vascular scaffold was engineered as a multilayer tubular construct consisting of an inner electrospun layer with fibers aligned in the axial direction (First layer), a second electrospun layer (Second layer) with fibers aligned circumferentially, followed by a central circumferentially aligned microfiber layer produced by 4‐axis printing (4‐axis layer), and an external electrospun layer (EEL) of randomly oriented nanofibers. These layers were fabricated sequentially as described below in the ‘Scaffold design’ section.

### 5.1 Chemicals and Reagents

Polycaprolactone (PCL, 80 kDa MW; Sigma‐Aldrich). Hexafluoro‐2‐propanol (HFIP; Biosolve B.V.). 1,6‐hexamethylenediamine (HMDA; Sigma‐Aldrich) 2‐propanol (Sigma‐Aldrich) *N*‐(3‐Dimethylaminopropyl)‐*N*′‐ethylcarbodiimide (EDC; Sigma‐Aldrich) and *N*‐Hydroxysuccinimide (NHS; Sigma‐Aldrich); 2‐(N‐Morpholino)ethanesulfonic acid (MES; Sigma‐Aldrich) buffer.

Toluidine Blue O (TBO) and Heparin sodium salt from porcine intestinal mucosa (Hep) were purchased from Sigma‐Aldrich. The marine sulfate polysaccharides were isolated from *Holothuria tubulosa* in our laboratory according to the previous reports [21, 22]. For in vitro testing, HUVECs (PromoCell; Cat. No. C‐12203) and CASMCs (Lonza Bioscience; Cat. No. CC‐2583) were cultured using Endothelial Cell Growth Medium (EGM‐2; Sigma‐Aldrich, Cat. No. C‐22011) and SmGM‐2 Smooth Muscle Cell Growth Medium‐2 BulletKit (Lonza Bioscience; Cat. No. CC‐3182), respectively. Xanthomonas campestris and Glycerol solution were purchased from Sigma‐Aldrich and used for mimicking blood rheology.

### 5.2 Scaffold Design: Production and Fabrication

For the first two electrospun layers (first layer and second layer), a polymer solution was prepared by dissolving 13% (w/v) polycaprolactone (PCL, 80 kDa MW) in hexafluoro‐2‐propanol (HFIP; Biosolve B.V., the Netherlands) under continuous stirring overnight. The solution was then electrospun using a blunt 20‐gauge stainless steel hypodermic needle (internal diameter 0.8 mm, Unimed). To ensure a homogeneous deposition the needle was set to move along the length of the collector with a speed of 30 mm/s. Electrospinning was performed under controlled conditions (temperature = 25°C, relative humidity = 35%) with the following parameters: an applied voltage of 18.5 kV, a needle‐to‐collector distance of 15 cm, and a flow rate of 1 mL/h. A stainless‐steel mandrel (60 mm diameter) was used as the collector, which rotation was set at 5800 rpm (Peripheral speed ≈ 18 m/s.) to obtain aligned fibers. During this process, nanofibers were collected on an aluminum foil placed on the collector for 45 min. Following deposition, the resulting PCL meshes were carefully rolled onto a 4 mm diameter, 10 cm long aluminum mandrel such that the inner layer had fibers aligned along the mandrel's axis, to allow the correct HUVECs orientation, while the second layer exhibited circumferential fiber alignment, to allow CASMCs orientation.

On top of the first 2 electrospun layers, a central circumferentially aligned microfiber layer was fabricated using a 4‐axis printing system [26, 63]. The preformed two‐layer electrospun construct, mounted on the 4 mm mandrel, was incorporated into this system. PCL (80 kDa MW) was melted at 150°C for 30 min in a heated metal cartridge and subsequently extruded through a 260 µm diameter nozzle (25G encapsulation needle, DL Technology) under a pressure of 7 bar. The mandrel, secured to a DC motor (Unimat 12 V DC powered by a Voltcraft LPS1153 power supply, Conrad), rotated at 1060 rpm (Peripheral speed ≈ 0.222 m/s) while the printhead traversed the collector at a speed of 0.75 mm/s over 10 passes, depositing the middle microfiber layer. This layer exhibited increased porosity, facilitating CASMC infiltration and alignment, while simultaneously offering protection against delamination of the inner layers.

Finally, the composite construct (comprising the two electrospun layers and the printed middle layer) was transferred back to the electrospinning system to form the outermost layer. Using the same PCL‐HFIP solution (13% w/v) as before, this final external electrospun layer (EEL) was obtained collecting the nanofibers for 15 min with the following settings: 18.5 kV applied voltage, 150 mm working distance, and 1 mL/h flow rate. The 4 mm mandrel speed was set at 1000 rpm (Peripheral speed ≈ 0.209 m/s) to produce randomly oriented fibers. This last step formed an external nanofibrous layer that reinforced the scaffold and further mimicked the native extracellular matrix of the vascular adventitia.

### 5.3 Scanning Electron Microscopy Investigation

For the characterization of fiber morphology and size, samples were first sputter‐coated with gold (Quorum Technologies SC7620). Scanning electron microscopy (SEM) images were then acquired using a JSM‐IT200 microscope (Jeol Ltd.) at varying magnifications, with an accelerating voltage of 100 kV and a working distance of 11 mm. Subsequent image processing and analysis were performed to quantify fiber diameter and orientation using the Fiji image processing package (GNU General Public License), based on measurements from three samples with 35 observations each.

The nanofiber orientation was investigated with the “Directionality” plugin of ImageJ [64]. This approach allowed us to quantify the number of nanofibers within a given angle range from the axis, using a “Local Gradient Orientation” method, following a previously validated procedure [65]. The analysis was performed on 3 images per layer (magnification = 1000×) along the different principal orientations of fibers with respect to the scaffold's axis and the results reported as mean and standard deviation.

### 5.4 Marine Sulfated Polysaccharides Purification and Quantification

Marine sulfated polysaccharides were purified following the protocol described by Nieddu et al. [21]. with minor modifications. In brief, body wall fragments from *Holothuria tubulosa* were sequentially treated with absolute ethanol and acetone to achieve dehydration and delipidation. The dehydrated tissues were rehydrated in 0.1 M sodium acetate buffer (pH 6.0) and subsequently digested with papain. After centrifugation, the supernatant was subjected to anion‐exchange chromatography on a DEAE‐Sephacel column, and the 1.5 M lithium chloride‐eluted fractions, referred to as Ht (*H. tubulosa*), were quantified for hexuronic acid (UA) content using the Bitter and Muir method [66].

### 5.5 Scaffold Functionalization via Aminolysis and EDC/NHS Crosslinking of Sulfated Polysaccharides

As previously described by Obino et al. [22], PCL scaffolds were functionalized using a two‐step protocol. In the first step, scaffolds underwent aminolysis by incubating with a 6% (w/v) solution of 1,6‐hexamethylenediamine (HMDA) in 2‐propanol at 37°C for 2 h. This treatment was followed by successive washes with 2‐propanol and distilled water to remove residual HMDA. In the subsequent step, the aminolyzed scaffolds were soaked in a solution containing 166 µg_UA_/mL of Ht or unfractionated heparin (Hep), prepared in 0.1 M EDC/NHS dissolved in 2‐(N‐morpholino)ethanesulfonic acid (MES) buffer at pH 5 and incubated for 24 h at room temperature, then thoroughly washed with PBS to eliminate any unreacted reagents. Following functionalization, scaffolds were classified into four groups: PCL, referring to untreated PCL scaffolds; PCL‐EDC/NHS, representing scaffolds subjected to aminolysis and EDC/NHS crosslinking in the absence of polysaccharides; PCL‐Hep, scaffolds functionalized with heparin; and PCL‐Ht, scaffolds functionalized with sulfated polysaccharides derived from *Holothuria tubulosa*.

### 5.6 Mechanical Characterization of Multilayered sTEVGs

Mechanical properties of multilayered tubular scaffolds were evaluated using an ElectroForce 3200 Series III mechanical tester (TA Instruments) equipped with a 45 N load cell. Circumferential tensile tests were conducted at a strain rate of 25%/s until sample failure following ISO 7198:2016 guidelines [67]. As clamping system, custom‐made capstan grips, consisting of two 1.5 mm‐diameter stainless steel circular pins were employed.

Scaffolds were cut into tubular segments of 5 mm length and tested after 24‐h PBS immersion at 37°C. The test was captured using a camera (DMC‐G3, Panasonic) with a macrolens (Panagor 90 mm f2.8, Komine).

#### 5.6.1 Scaffold Configuration Tested

Various scaffold configurations were evaluated to assess the contribution of each layer to the overall performance. The following individual and combined layers were tested: (i) the first electrospun layer alone (first layer), (ii) the second electrospun layer alone (second layer), (iii) the 4‐axis printed layer (4‐axis) combined with the electrospun external layer (EEL), and (iv) the full layered construct (MultiLayer). Additional configurations are presented in the supplementary information and include: the sTEVG without the 4‐axis layer, and a sTEVG with a duplicated second electrospun layer. Furthermore, the fully assembled four‐layer scaffold was also evaluated after surface functionalization. The tested functionalized conditions included: unmodified PCL (PCL), PCL activated with EDC/NHS chemistry (PCL–EDC/NHS), PCL functionalized with heparin (PCL–Hep), and PCL functionalized with sulfate polysaccharides from *Holothuria tubulosa* (PCL–Ht).

#### 5.6.2 Dimensional Analysis

Wall thickness measurements were acquired using a Nikon SMZ25 automated stereomicroscope with ring LED illumination. For each scaffold, 20 equidistant thickness measurements were taken from high‐resolution images (5× magnification) and analyzed via ImageJ (v1.53), with results expressed as mean ± standard deviation.

#### 5.6.3 Mechanical Parameter Calculation

The mechanical properties of scaffolds were calculated adopting two different approaches. At first, the apparent stress (σ_a_), accounting only the geometrical properties of scaffolds, was obtained as follows:

(1) $$\sigma =\frac{F}{A}$$

where F is the force (N) recorded and A the area (mm^2^), defined as:

(2) A=2·l·t

where l is the width (mm^2^) and t the wall thickness (mm^2^) of the tested tubular scaffold.

The second consolidated method was the evaluation of the net stress (σ_n_), which allows to evaluate the mechanical properties of the specimen avoiding the contribution of its internal porosity [68, 69, 70, 71]. The net stress was calculated by dividing the apparent stress by the volume fraction (*v*) of the specimens. The *v* was calculated by using the equation:

(3) $$\upsilon =\frac{w}{\left(L\cdot A\cdot \rho \right)}$$

where w is the weight of the specimen (g), L is the gauge length of the specimen (mm), A is the area of the specimen as previously defined, ρ is the density of PCL (ρPCL = 1.145 g/cm^3^). The weight of each specimen was calculated using a precision balance (MG214Ai‐M, VWR International, Radnor, USA). The following indicators were considered: Yield Force (F_Y_), Yield Stress (σ_Y_), Yield Strain (ε_Y_), Elastic Modulus (E), Failure Force (F_F_), Failure Stress (σ_F_), Failure Strain (ε_F_), Toughness to Yield (W_Y_), Toughness to Failure (W_F_) (these last two values calculated by using the trapezoid method as the integral of the area under the stress‐strain curves).

To calculate the elastic modulus, a procedure adapted from a well‐established method was employed [68]. Briefly, the initial toe region, corresponding to the first 5% of the failure stress, was excluded from each stress–strain curve to ensure comparability across samples and to mark the onset of the linear elastic region. This step avoids including into the calculation of the elastic modulus, the initial toe‐region where the nanofibers are recovering from their wavy‐shape before the application of the load. Next, a preliminary yield point was visually estimated, and an initial linear regression was performed on the region extending from the start of the linear region to 50% of the strain span between this point and the tentative yield point. To minimize operator bias, a second line parallel to this regression was offset by 2% strain. The official yield point was then defined as the intersection between this offset line and the stress–strain curve. The elastic modulus was calculated as the slope of a second linear regression between the start of the linear region and the official yield point.

As far as σ_Y_ and yield strain (ε_Y_) are concerned, they were determined by evaluating the meeting point between the stress‐strain curve and a straight line, having modulus equal to the calculated elastic modulus, horizontally shifted by 2% with respect to the linear section of the stress‐strain curve. For all experiments, five specimens per condition were analyzed.

The volume fraction of each layer and of the whole scaffolds also allowed the calculation of the percentage porosity (P) of samples as follows:

(4) $$P\left(\%\right)=(1-v)\cdot 100$$

#### 5.6.4 Compliance Estimation From Pressure–Strain Curves

Compliance was estimated from the low‐pressure region of the pressure–strain curves, specifically within the physiological range typical of human arteries (80–120 mmHg). For each construct, the strain values at 80 mmHg and 120 mmHg were extracted directly from the plots. Compliance was then calculated as the change in strain (Δε) divided by the corresponding change in pressure (ΔP), following the expression:

(5) $$Compliance=\frac{\Delta \varepsilon }{\Delta P}=\left(\frac{\varepsilon 120-\varepsilon 80}{120\;mmHg-80\;mmHg}\right)x100$$

This formulation is derived from the classical definition of vascular compliance:

(6) $$Compliance=\frac{\Delta D/D0\varepsilon }{\Delta P}$$

where 𝐷*_0_* is the initial diameter and Δ𝐷 is the change in diameter due to pressurization. Under the assumption of small deformations and uniform radial expansion, circumferential strain (𝜀) can be approximated as Δ𝐷/𝐷*_0_*. Therefore, strain per unit pressure serves as a valid proxy for relative compliance. The resulting compliance values are normalized and expressed in terms of percent strain per mmHg (%mmHg) over the 80–120 mmHg range.

#### 5.6.5 Burst Pressure Analysis

Direct burst pressure measurements were conducted using a custom‐built testing platform designed for direct evaluation of scaffold mechanical integrity. Tubular scaffolds (length: 30 mm) were firmly mounted between two PTFE connectors (internal diameter: 3 mm). To ensure a leak‐free seal, the scaffold ends were secured with rubber bands. The assembled system was incorporated into a closed‐loop fluidic circuit for a real‐time pressure monitoring, which included a microfluidic flow controller (OB1 MK4 – ELVEFLOW) and a high‐precision liquid mass flow sensor (ELVEFLOW; flow range: 40–1000 µL/min; maximum pressure: 15 bar).

The system delivered a controlled, stepwise increase in internal pressure through the fluidic circuit. Pressure was continuously recorded until scaffold failure (burst event), which was both visually confirmed and captured using a high‐resolution camera (Panasonic DMC‐G3, The Netherlands) equipped with a Panagor 80 mm f/2.8 macro lens (Komine, Japan).

To closely replicate the hemodynamic conditions and non‐Newtonian rheology of blood, a blood‐mimicking fluid was prepared with the following composition: 0.075% (w/v) xanthan gum (XG), 50% (v/v) distilled water, and 50% (v/v) glycerol (Sigma Aldrich, US). This formulation was selected based on literature demonstrating its ability to approximate both the viscosity (3.5–4.5 mPa·s) and shear‐thinning behavior characteristic of human blood, particularly at varying shear rates and vessel diameters [72, 73]. The addition of xanthan gum was essential to reproduce the non‐Newtonian properties of blood, wherein viscosity increases at lower shear rates due to red blood cell aggregation, a phenomenon critical for physiological relevance in small vessel testing. To increase the visual contrast during perfusion and pressure testing, a red food colorant was added to the solution.

Pressure data were acquired continuously using the ELVEFLOW Smart Interface (ESI) software and the burst pressure was defined as the maximum pressure recorded immediately prior to scaffold rupture. Each scaffold type was tested in quintuplicate (n = 5), and results were reported as mean ± standard deviation.

In parallel, an indirect estimation of burst pressure was performed by applying a modified form of Laplace's law, which relates the tensile rupture force to internal pressure in a thin‐walled cylindrical geometry, using the following definition, as already reported [74].

(7) $$BP=\frac{F\left(N\right)}{L_{0}Df}$$

where F is the failure force measured in tensile testing, 𝐿_0_ is the initial unloaded internal diameter of the scaffold, and 𝐷𝑓 is the internal diameter at the point of failure. This indirect method allows for comparison between pressurization‐induced rupture and tensile‐derived mechanical performance, providing complementary validation of scaffold strength.

### 5.7 Spectroscopic, Colorimetric, and Elemental Characterization of Scaffold Functionalization

The chemical composition and surface functionalization of the electrospun scaffolds were characterized using Attenuated Total Reflectance Fourier Transform Infrared Spectroscopy (ATR‐FTIR) spectroscopy, Toluidine Blue O (TBO) assay, and energy‐dispersive X‐ray spectroscopy (EDX).

#### 5.7.1 ATR‐FTIR Analysis

ATR‐FTIR analysis was performed using a Nicolet iS50 spectrometer (Thermo Fisher Scientific, the Netherlands) equipped with an attenuated total reflectance (ATR) module. Spectra were collected in the 400–4000 cm^−1^ range at a resolution of 0.5 cm^−1^, averaging 32 scans per sample. Prior to measurement, scaffolds were cut into sections and pressed against the ATR crystal to ensure optimal contact. Background spectra were recorded under identical conditions and subtracted to minimize atmospheric interference. FTIR analysis served to verify the presence of functional groups related to the crosslinking chemistry. These data were consistent with a previously published analysis, which confirmed successful crosslinking of sulfated polysaccharides to PCL‐based scaffolds [22].

#### 5.7.2 Toluidine Blue O Assay

Quantification of sulfated polysaccharide incorporation was carried out using a modified Toluidine Blue O (TBO) assay. Scaffolds (PCL‐Ht, PCL‐Hep, and PCL‐EDC/NHS as control) were incubated in a TBO working solution (0.04% w/v in 0.01 M HCl, 0.2 M NaCl) under agitation for 1 h. After incubation, samples were washed five times for 10 min with 0.2 M NaCl to remove unbound dye and subsequently transferred to an elution solution (50% ethanol in 0.01 M HCl) for complete dye extraction overnight. The eluted dye was quantified by measuring absorbance at 630 nm using a CLARIOstar Plus plate reader. For standard curve preparation, 1 mL of the TBO working solution was combined with 100 µL of a Hep solution of known concentrations (0 µg/mL; 0.5 µg/mL; 1 µg/mL; 2.5 µg/mL; 5 µg/mL). This mixture was incubated at 37°C with gentle shaking for 1 h, during which the Hep–TBO complex spontaneously formed and precipitated. The mixture was then centrifuged at 300 rcf for 10 min, after which the supernatant was discarded. The precipitate was carefully rinsed five times with the washing solution to ensure complete removal of any unbound TBO. Finally, 1 mL of the elution solution was added to dissolve the precipitate, releasing the bound TBO, and the absorbance of the resulting solution was measured at 630 nm. Before the elution step, scaffolds were imaged using a SMZ25 stereomicroscope (Nikon Instruments, Japan) with an episcopic LED illumination ring to provide uniform reflected light.

#### 5.7.3 Energy‐Dispersive X‐ray Spectroscopy

Elemental analysis was performed using EDX to detect the presence of sulfur as an indicator of sulfated polysaccharide incorporation. Scaffold samples were mounted on stubs, sputter‐coated with a conductive layer of carbon (EM ACE600 High Vacuum Sputter Coater—Leica Microsystems), and analyzed using the JSM‐IT200 microscope. PCL‐Ht and PCL‐Hep scaffolds were compared to unmodified PCL to confirm the presence of sulfur atoms associated with sulfate groups introduced during functionalization.

### 5.8 HUVECs Static Seeding and Culture

HUVECs were obtained from PromoCell GmbH (Cat. No. C‐12203) and cultured in Endothelial Cell Growth Medium (EGM‐2, Sigma‐Aldrich, Cat. No. C‐22011) following the manufacturer's guidelines. Tubular sTEVGs were fabricated with a 4 mm internal diameter and sectioned into 5 mm segments. To ensure sterilization, scaffolds were immersed in 70% ethanol three times for 15 min each, air‐dried overnight, and subsequently rinsed with phosphate‐buffered saline (PBS). HUVECs were then seeded onto the luminal surface of the scaffolds at a density of 2 × 10^6^ cells/mL. The cell‐seeded constructs were maintained under static conditions in a humidified incubator at 37°C and 5% CO_2_, with the culture medium refreshed every 48 h. At defined time points (days 1, 4, 7, 14, and 21 post‐seeding), assessments of cell metabolic activity, proliferation, and morphology were performed to evaluate scaffold biocompatibility and cellular response.

### 5.9 HUVECs Dynamic Culture

For dynamic culture, sTEVGs were fabricated with a 4 mm internal diameter and sectioned into 30 mm segments, sterilized and positioned within a custom‐built bioreactor chamber. HUVECs were seeded into the luminal surface at the cell density of 2 × 10^6^ cells/mL. To promote uniform cell distribution, the chambers were rotated of 90° every 20 min during the first 2 h and every hour for additional 4 h. Then, cells were allowed to adhere for 24 h under static conditions. Following this initial seeding phase, the bioreactor chamber was connected to a peristaltic pump, which applied a continuous flow of culture medium at a rate of 1 mL/min (Shear stress ≈ 0.027 dyn/cm^2^) for 7 days.

### 5.10 CASMCs Static Seeding and Culture

Human coronary artery smooth muscle cells (CASMCs) were obtained from Lonza Bioscience (Cat. No. CC‐2583) and cultured in SmGM‐2 Smooth Muscle Cell Growth Medium‐2 BulletKit (Lonza Bioscience, Cat. No. CC‐3182). sTEVGs scaffolds, featuring a 4 mm internal diameter, were fabricated, sectioned into tubular segments of 5 mm length and placed in Eppendorf tubes of 0.5 mL. Then, they were sterilized with 70% ethanol washing, rinsed with PBS and placed in 48 multi well plates. To prevent unwanted colonization of the lumen, the scaffolds were filled with a 1.5% agarose gel. CASMCs were then seeded on the scaffolds at a density of 6 × 10^5^ cells/mL by adding 1 mL of cell suspension to each scaffold. To promote uniform cell distribution, the tubes were rotated 90° every 20 min during the first 2 h and every hour for additional 4 h. Following seeding, the scaffolds were transferred to non‐treated 48‐well plates for overnight incubation; the next day, constructs were relocated to fresh plates to remove any cells that had adhered to the well surfaces. Finally, the CASMC‐seeded scaffolds were maintained under static conditions in a humidified incubator at 37°C and 5% CO_2_ for 21 days, with medium changes occurring every 48 h.

### 5.11 Statistical Analysis

Statistical analysis was performed using GraphPad Prism version 10.0. Results are reported as mean ± standard deviation of at least three independent experiments. Differences among multiple groups at various time points were assessed using a 2‐way ANOVA analysis followed by Tukey's multiple comparisons test (DNA analysis). An ordinary one‐way ANOVA followed by Tukey's multiple comparisons test was conducted on mechanical characterization, burst pressure and compliance data. For the stress–strain curves, the median curve was used for graphical representation.

A *p*‐value of less than 0.05 was considered statistically significant (*p‐value < 0.05; **p‐value < 0.01; ***p‐value < 0.001; ****p‐value < 0.0001).

## Author Contributions

Draft, Writing Review & Editing, Visualization. A.S.: Conceptualization, Methodology, Supervision, Investigation, Formal analysis Validation, Writing Review & Editing. T.t.B.: Methodology, Investigation, Formal analysis, Visualization, Writing Review & Editing. G.N.: Conceptualization, Validation. T.B.: Methodology. G.A.D.: Funding acquisition. M.v.G.: Supervision, Resources. M.F.: Supervision, Funding acquisition, Resources. A.J.L.: Conceptualization, Supervision, Writing Review & Editing, Funding acquisition. L.M.: Conceptualization, Supervision, Writing Review & Editing, Funding acquisition, Project. administration.

## Conflicts of Interest

The authors declare no conflict of interest

## Supporting information




**Supporting File 1**: adhm70862‐sup‐0001‐SuppMat.docx.

**Supporting File 2**: adhm70862‐sup‐0002‐V1.mp4.

**Supporting File 3**: adhm70862‐sup‐0003‐V2.mp4.

**Supporting File 4**: adhm70862‐sup‐0004‐V3_Day1 PCL CASMCs.

**Supporting File 5**: adhm70862‐sup‐0005‐V4_Day1 HT CASMCs.gif.

**Supporting File 6**: adhm70862‐sup‐0006‐V5_Day 1 Hep CASMCs.gif.

## Data Availability

The data that support the findings of this study are available from the corresponding author upon reasonable request.
