# Supplementary material for: Tunable Bioresorbable Scaffolds With Marine Sulfated Polysaccharides for Small‐Caliber Vascular Grafts: A Multi‐Layered Strategy Combining Electrospinning and 4‐Axis Printing
Source: Adv Healthc Mater. 2026 Feb 2;15(16):e05314. doi: 10.1002/adhm.202505314 (PMC13107931; doi:10.1002/adhm.202505314)
Supplement: Supplementary file 1 — Supporting File 1: adhm70862‐sup‐0001‐SuppMat.docx. [file ADHM-15-0-s001.docx]

# Supplementary Information

**Tunable Bioresorbable Scaffolds with Marine Sulfated Polysaccharides for Small-Caliber Vascular Grafts: A Multi-Layered Strategy Combining Electrospinning and 4-Axis Printing**

Gabriele Obino^a,b^; Alberto Sensini^b,c^; Tim ten Brink^b^ ; Gabriele Nieddu^a^; Tristan Bodet^b^; Giovanni Andrea Deiana^d^; Martijn van Griensven^c^; Marilena Formato^a^; Antonio J Lepedda^a **^ and Lorenzo Moroni^b **^

^a^Department of Biomedical Sciences, University of Sassari, Viale San Pietro, 43b, 07100 Sassari, Italy

^b^ Department of Complex Tissue Regeneration, MERLN Institute for Technology-Inspired Regenerative Medicine, Maastricht University, 6200 MD Maastricht, The Netherlands

^c^ Department of Cell Biology-Inspired Tissue Engineering, MERLN Institute for Technology-Inspired Regenerative Medicine, Maastricht University, 6200 MD Maastricht, The Netherlands

^d^Department of Medicine, Surgery and Pharmacy, University of Sassari, Viale San Pietro, 43b, 07100 Sassari, Italy

**Corresponding authors:**

Lorenzo Moroni

e-mail: [l.moroni@maastrichtuniversity.nl](mailto:l.moroni@maastrichtuniversity.nl)

**Lead Contact**:

Lorenzo Moroni

e-mail: [l.moroni@maastrichtuniversity.nl](mailto:l.moroni@maastrichtuniversity.nl)


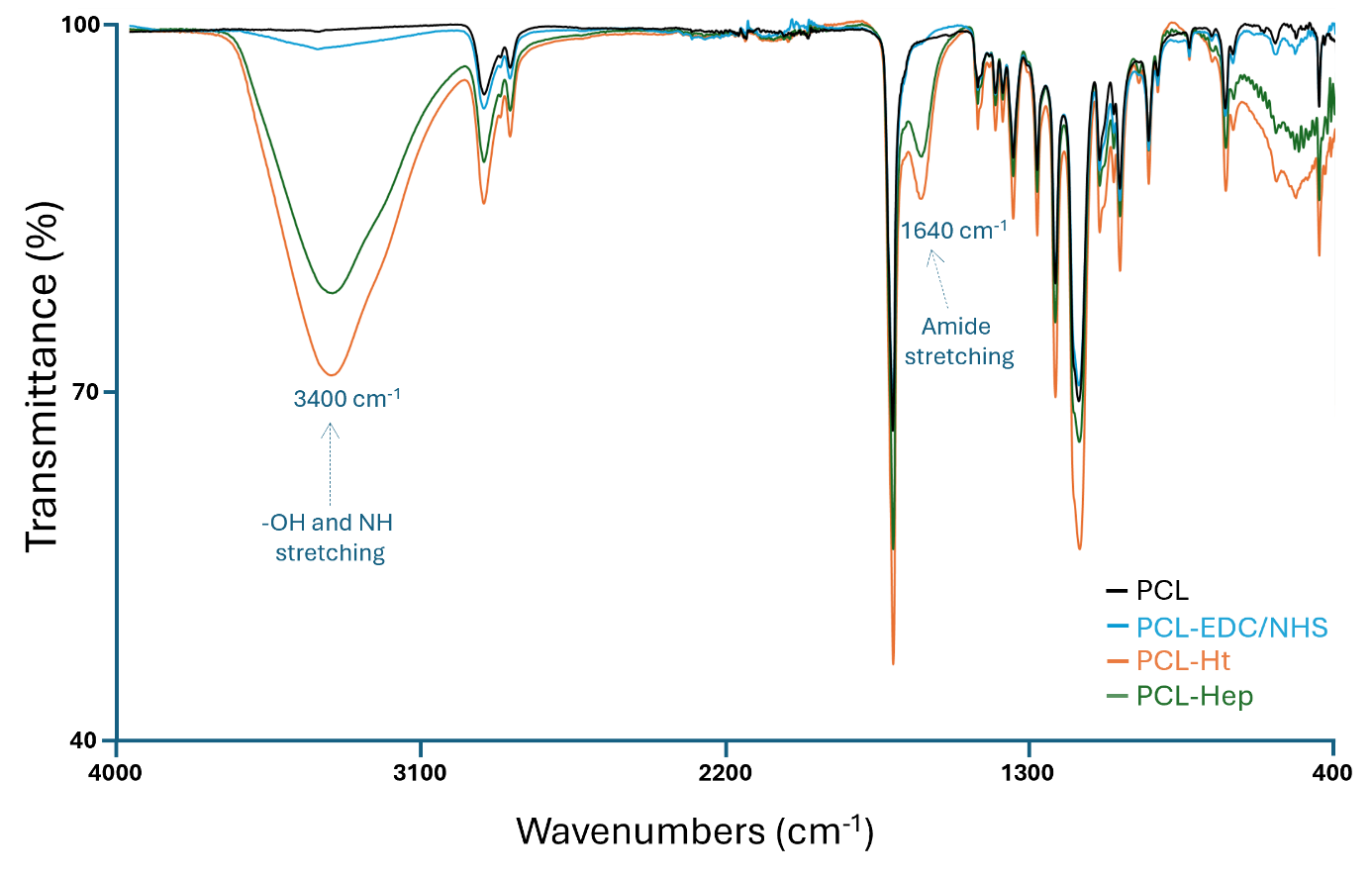


**Figure S1.** ATR-FTIR spectra of naked PCL, aminolyzed PCL treated with EDC/NHS (PCL-EDC/NHS), PCL functionalized with Hep (PCL-Hep), or with polysaccharides from Ht (PCL-Ht).


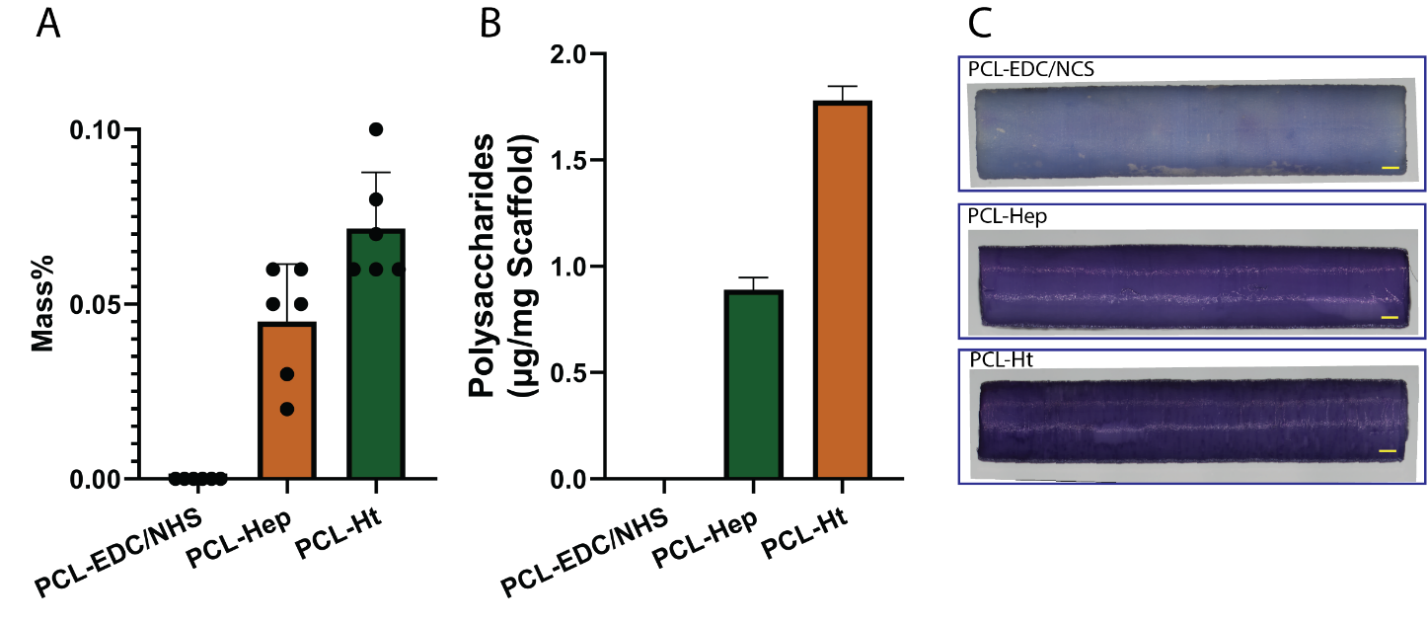


**Figure S2.** Characterization of scaffold functionalization through EDX (A), TBO assay quantification (B) and TBO qualitative staining (C). Scale bar 1mm.


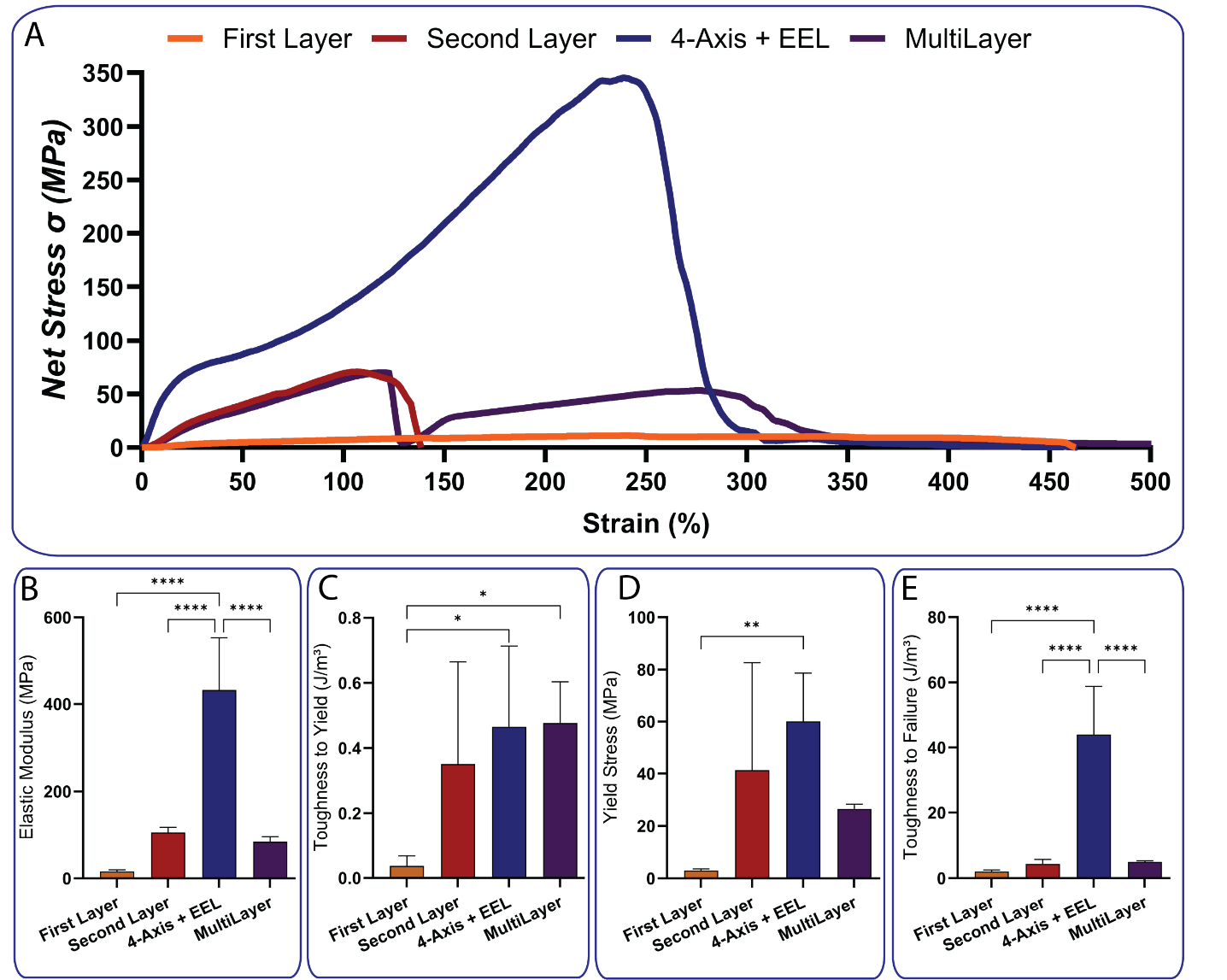


**Figure S3.** Mechanical characterization of multilayered TEVGs. (A) Representative net stress-strain curves for scaffolds with increasing numbers of layers. (B) Net elastic modulus, (C) net toughness to yield, (D) net yield stress and (E) net toughness to failure for each scaffold configuration. Data: mean ± SD (n=5). Statistics: ordinary one-way ANOVA followed by Tukey’s multiple comparisons test. *p-value < 0.05; **p-value < 0.01; ****p-value < 0.0001.


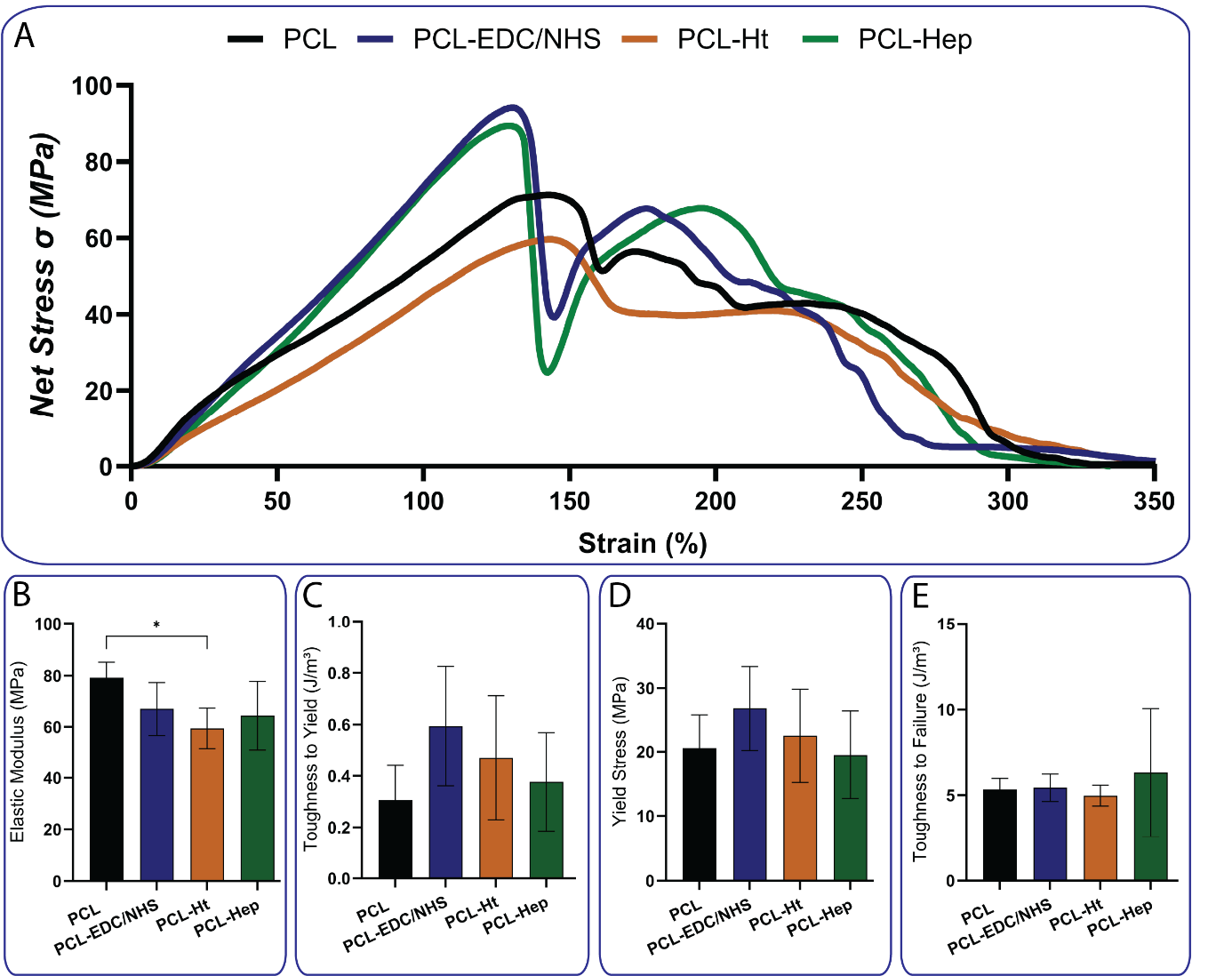


**Figure S4.** Mechanical properties of functionalized TEVGs. (A) Representative net stress–strain curves for PCL, PCL–EDC/NHS, PCL–Ht, and PCL–Hep scaffolds. (B) Net elastic modulus. (C) net toughness to yield (D) net yield stress, and (E) net toughness to failure for each scaffold functionalization. Data: mean ± SD (n=5). Statistics: ordinary one-way ANOVA followed by Tukey’s multiple comparisons test. *p-value < 0.05.


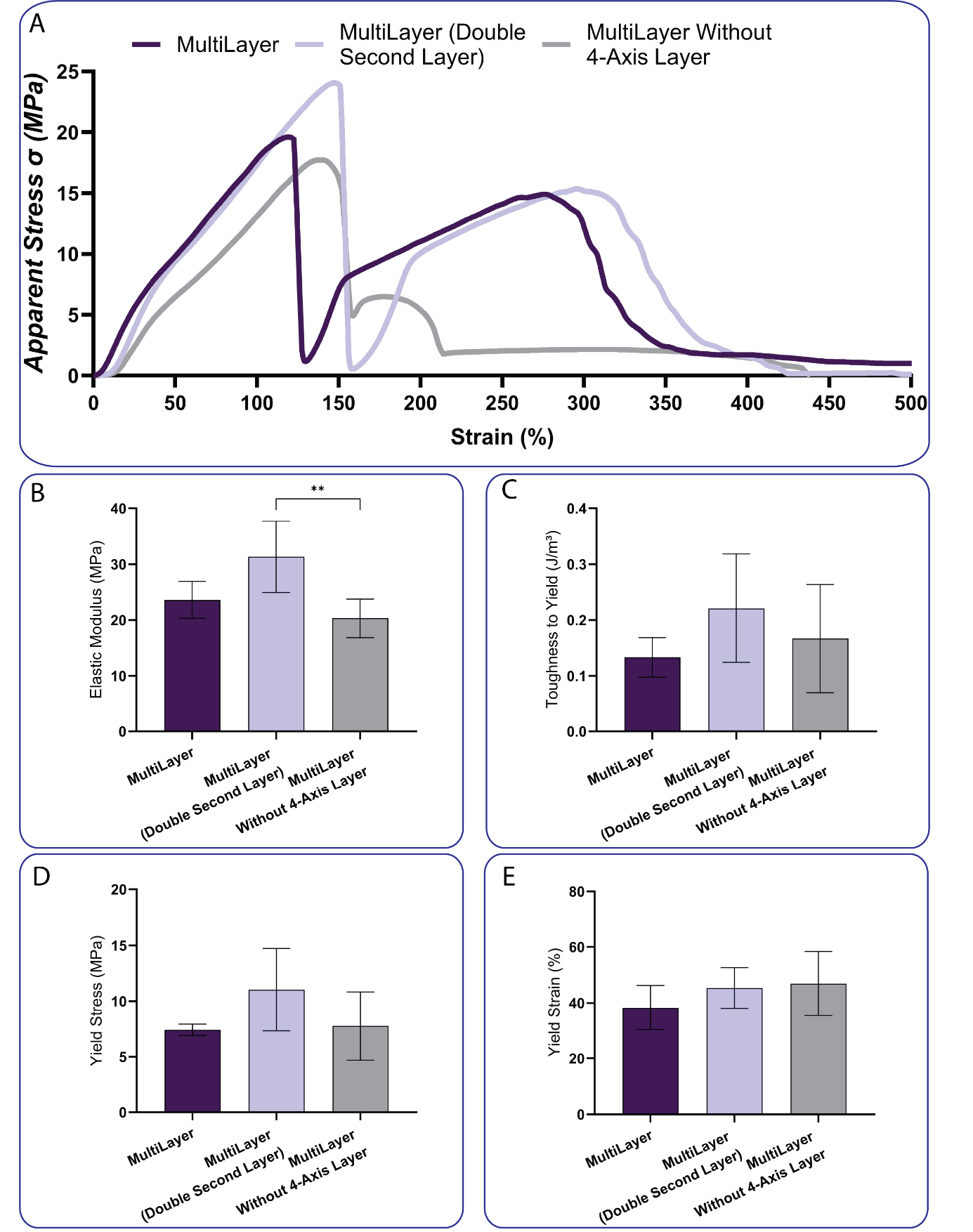


**Figure S5.** Mechanical tuning of the multilayer TEVG through scaffold design. (A) Representative stress–strain curves for scaffolds with different layer configurations, showing the effect of adding or omitting specific layers, (B) elastic modulus across configurations, (C) apparent toughness to yield, (D) apparent yield stress (E) and yield strain of each configuration. Data: mean ± SD (n=5). Statistics: ordinary one-way ANOVA followed by Tukey’s multiple comparisons test. **p-value < 0.01.


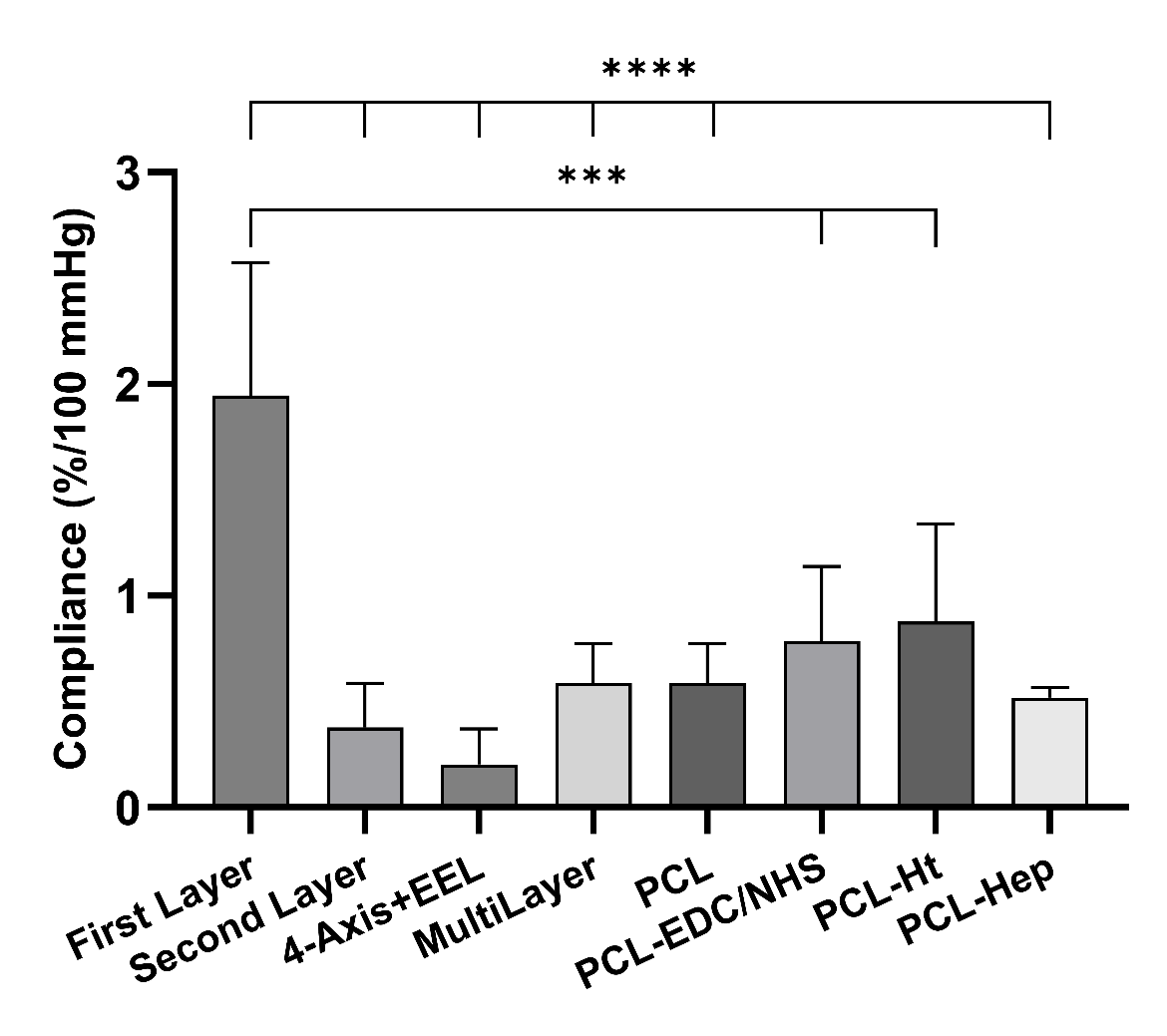


**Figure S6.** Compliance of the different layers and multilayer conformations. Data: mean ± SD (n=5). Statistics: ordinary one-way ANOVA followed by Tukey’s multiple comparisons test. ***p-value < 0.001; ****p-value < 0.0001


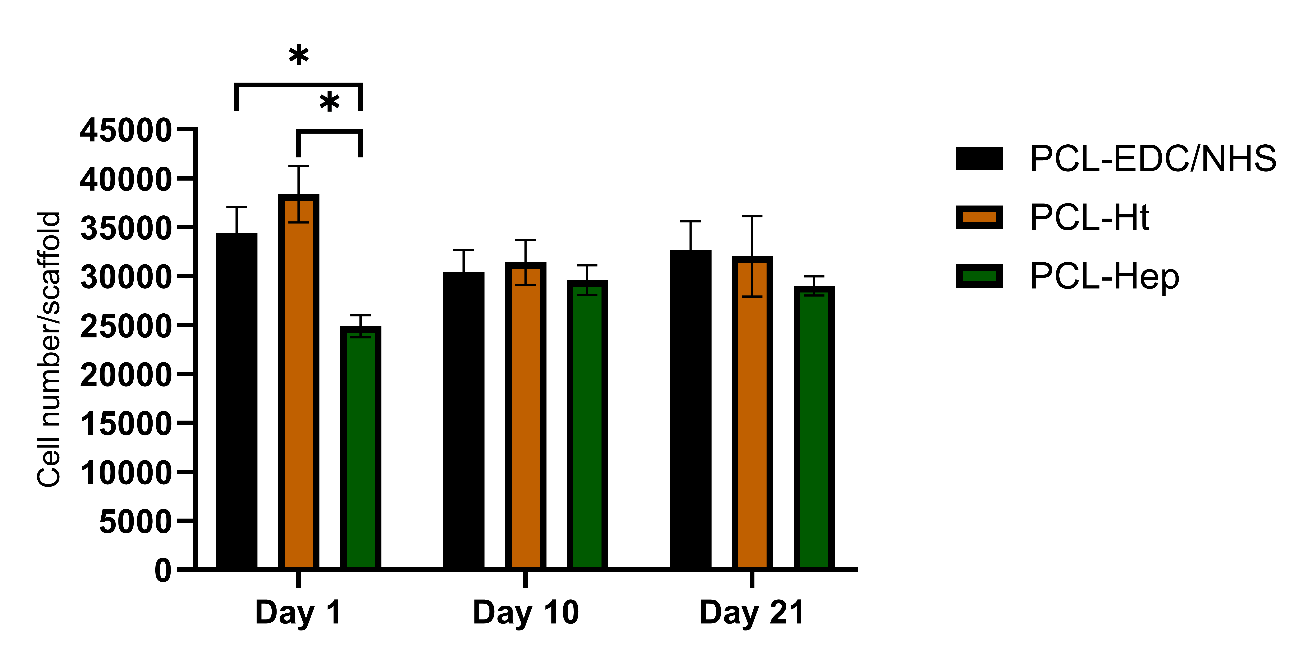


**Figure S7.** DNA quantification of HUVECs, expressed in cell number per scaffold, on PCL-EDC/NHS, PCL-Ht, and PCL-Hep scaffolds at days 1, 10, and 21. Data: mean ± SD (n=3). Statistics: 2-way ANOVA analysis followed by Tukey’s multiple comparisons test. *p-value < 0.05.


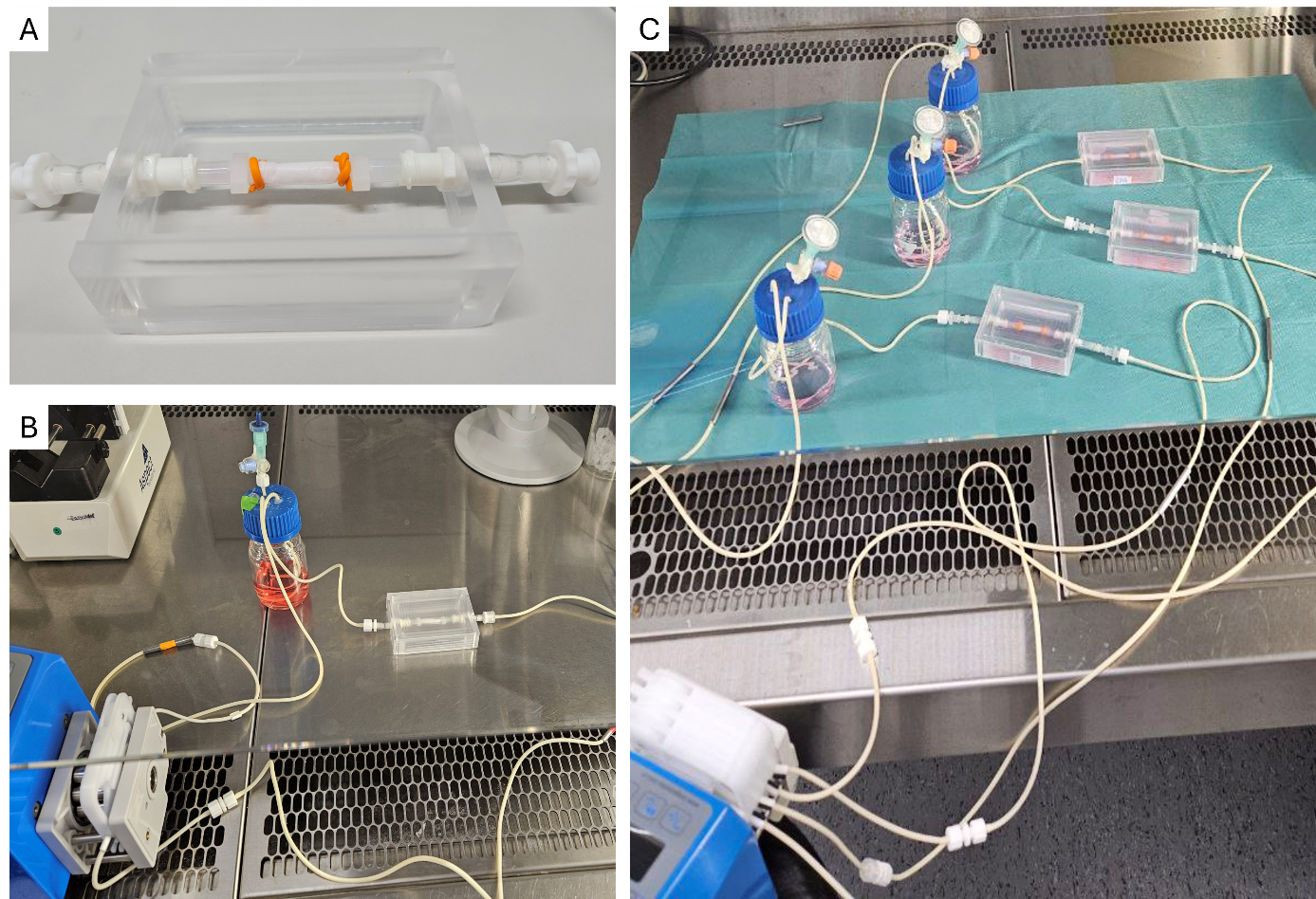


**Figure S8.** **Custom-built bioreactor system for HUVEC dynamic culture. A**) Chamber design for tubular scaffold mounting. B-C) Complete perfusion setup integrating culture medium reservoirs, peristaltic pump, and bioreactor chambers for parallel dynamic culture experiments.


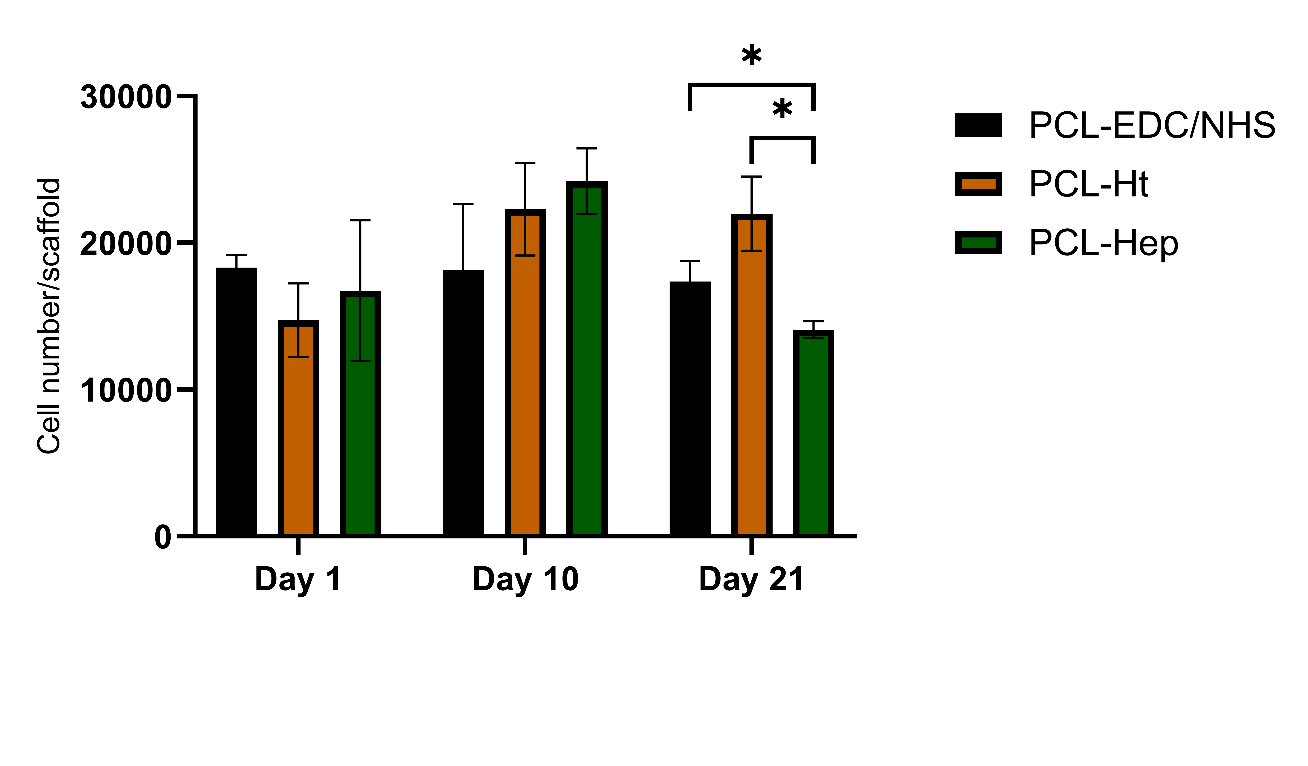


**Figure S9.** DNA quantification of CASMCs, expressed in cell number per scaffold, on PCL-EDC/NHS, PCL-Ht, and PCL-Hep scaffolds at days 1, 10, and 21. Data: mean ± SD (n=3). Statistics: 2-way ANOVA analysis followed by Tukey’s multiple comparisons test. *p-value < 0.05.

|  | Failure Force  (N) | Failure Strain  (%) | Failure Stress  (N/mm^3^) | Yield stress  (MPa) | Yield strain  (%) | Elastic Modulus (MPa) | Thoughness to Yield (J/m^3^) | Thoughness to Failure (J/m^3^) |
| --- | --- | --- | --- | --- | --- | --- | --- | --- |
| First Layer | 3.9 ± 0.2 | 270.3 ± 32.4 | 3.9 ± 0.2 | 1.1 ± 0.2 | 26.3 ± 12.1 | 5.6 ± 1.2 | 0.013 ± 0.011 | 0.037 ± 0.031 |
| Second Layer | 23.5 ± 7.1 | 116.5 ± 8.1 | 24.4 ± 7.4 | 15.2 ± 15.7 | 29.4 ± 9.9 | 38.6 ± 4.6 | 0.127 ± 0.113 | 0.351 ± 0.313 |
| 4-Axis+EEL | 34.9 ± 10.1 | 236.6 ± 22.8 | 33.9 ± 9.8 | 5.7 ± 1.8 | 17.6 ± 3.4 | 38.4 ± 12.6 | 0.044 ± 0.023 | 0.465 ± 0.247 |
| MultiLayer | 63.6 ± 3.3 | 127.1 ± 8.2 | 20.3 ± 1.0 | 7.4 ± 0.5 | 38.3 ± 7.9 | 23.6 ± 3.4 | 0.133 ± 0.036 | 0.476 ± 0.127 |
| PCL | 64.2 ± 6.8 | 137.1 ± 8.9 | 20.5 ± 2.2 | 5.8 ± 1.4 | 31.5 ± 4.7 | 22.2 ± 1.7 | 0.086 ± 0.038 | 0.306 ± 0.137 |
| PCL-EDC/NHS | 70.3 ± 13.0 | 134.9 ± 4.5 | 22.6 ± 3.9 | 7.5 ± 1.8 | 46.7 ± 10.0 | 18.7 ± 2.9 | 0.166 ± 0.065 | 0.593 ± 0.232 |
| PCL-Ht | 64.0 ± 9.6 | 136.7 ± 4.8 | 20.4 ± 3.1 | 6.3 ± 2.0 | 44.2 ± 9.6 | 16.6 ± 2.2 | 0.132 ± 0.067 | 0.471 ± 0.241 |
| PCL-Hep | 66.6 ± 18.8 | 129.0 ± 2.2 | 21.3 ± 6.0 | 5.5 ± 1.9 | 38.5 ± 6.8 | 18.0 ± 3.7 | 0.105 ± 0.054 | 0.376 ± 0.191 |
| Multilayer Double 2 Layer | 104.3 ± 27.1 | 134.0 ± 20.7 | 28.6 ± 7.4 | 11.0 ± 3.7 | 45.4 ± 7.3 | 31.4 ± 6.4 | 0.221 ± 0.097 | 0.660 ± 0.290 |
| Multilayer Without 4-Axis | 44.5 ± 14.3 | 134.9 ± 3.9 | 22.2 ± 7.2 | 7.8 ± 3.1 | 46.9 ± 11.5 | 20.3 ± 3.5 | 0.167 ± 0.097 | 0.429 ± 0.249 |

**Table S1.** Apparent mechanical properties of the scaffolds evaluated under uniaxial tensile testing. Parameters reported include failure force (F_F_), failure strain (ε_F_), failure stress (σF), yield stress (σ_Y_), yield strain (ε_Y_), elastic modulus (E), toughness to yield (W_Y_), and toughness to failure (W_F_). Data are expressed as mean (M) ± standard deviation (SD).

|  | Failure Stress  (N/mm^3^) | Yield stress  (MPa) | Elastic Modulus (MPa) | Thoughness to Yield (J/m^3^) | Thoughness to Failure (J/m^3^) | Volume Fraction | Porosity (%) |
| --- | --- | --- | --- | --- | --- | --- | --- |
| First Layer | 11.3 ± 0.7 | 3 ± 0.6 | 16 ± 3.6 | 0.718 ± 0.166 | 2.057 ± 0.477 | 0.35 | 65.1 |
| Second Layer | 67.6 ± 20.5 | 41.2 ± 41.3 | 89.1 ± 36.8 | 1.585 ± 0.488 | 4.402 ± 1.355 | 0.36 | 64 |
| 4-Axis+EEL | 359 ± 103 | 60.1 ± 18.6 | 433.4 ± 119.7 | 4.146 ± 1.401 | 43.939 ± 14.847 | 0.094 | 90.57 |
| MultiLayer | 72.6 ± 3.7 | 26.5 ± 1.8 | 84.5 ± 12.0 | 1.405 ± 0.096 | 5.021 ± 0.342 | 0.28 | 72.02 |
| PCL | 73.2 ± 7.8 | 20.6 ± 5.1 | 79.3 ± 6.1 | 1.492 ± 0.181 | 5.331 ± 0.646 | 0.28 | 72.02 |
| PCL-EDC/NHS | 80.8 ± 14.0 | 26.8 ± 6.6 | 67.0 ± 10.4 | 1.521 ± 0.222 | 5.436 ± 0.793 | 0.28 | 72.02 |
| PCL-Ht | 73.1 ± 10.9 | 22.5 ± 7.3 | 59.4 ± 8.0 | 1.393 ± 0.171 | 4.978 ± 0.610 | 0.28 | 72.02 |
| PCL-Hep | 76.1 ± 21.5 | 19.5 ± 6.8 | 64.4 ± 13.2 | 1.768 ± 1.045 | 6.318 ± 3.736 | 0.28 | 72.02 |
| Multilayer Double 2 Layer | 85.3 ± 22.1 | 32.9 ± 11.1 | 93.5 ± 19.0 | 1.944 ± 0.486 | 5.800 ± 1.449 | 0.34 | 66.5 |
| Multilayer Without 4-Axis | 57.1 ± 18.4 | 19.9 ± 7.9 | 51.6 ± 8.8 | 1.496 ± 0.453 | 3.843 ± 1.163 | 0.39 | 61.1 |

**Table S2.** Net mechanical properties of the scaffolds normalized for the material volume fraction (ν). Reported values include net failure stress, net yield stress, net elastic modulus, net toughness to yield, and net toughness to failure. Data are presented as mean (M) ± standard deviation (SD). Volume fraction and porosity were also reported as single value per each scaffold conformation.

**Video S1.** Representative tensile test video of the tubular scaffold under uniaxial loading. The video shows the progressive elongation and deformation of the sample over time during tensile testing, from the initial undeformed state to failure.

**Video S2.** Representative burst pressure test video of the vascular scaffold. The video shows the gradual pressurization of the scaffold over time, from the initial unpressurized state to burst.

**Video S3.** Representative z-stack immunofluorescence video of CASMCs cultured under static conditions on the external surface of non-functionalized PCL tubular scaffolds. The video shows cellular distribution and organization across the scaffold thickness, with CASMCs seeded on the microfiber outer layer. Red fluorescence indicates α-smooth muscle actin (α-SMA), while blue fluorescence (DAPI) marks cell nuclei. Images were acquired at 25× magnification. Scale bar: 50 μm

**Video S4.** Representative z-stack immunofluorescence video of CASMCs cultured under static conditions on the external surface of PCL–Ht tubular scaffolds. The video illustrates cellular distribution and organization across the scaffold thickness, with CASMCs seeded on the microfiber outer layer. Red fluorescence indicates α-smooth muscle actin (α-SMA), while blue fluorescence (DAPI) marks cell nuclei. Images were acquired at 25× magnification. Scale bar: 50 μm.

**Videos S5.** Representative z-stack immunofluorescence video of CASMCs cultured under static conditions on the external surface of PCL–Hep tubular scaffolds. The video illustrates cellular distribution and organization across the scaffold thickness, with CASMCs seeded on the microfiber outer layer. Red fluorescence indicates α-smooth muscle actin (α-SMA), while blue fluorescence (DAPI) marks cell nuclei. Images were acquired at 25× magnification. Scale bar: 50 μm.
