# Supplementary figures and images for: Tunable Bioresorbable Scaffolds With Marine Sulfated Polysaccharides for Small‐Caliber Vascular Grafts: A Multi‐Layered Strategy Combining Electrospinning and 4‐Axis Printing
Source: Adv Healthc Mater. 2026 Feb 2;15(16):e05314. doi: 10.1002/adhm.202505314 (PMC13107931; doi:10.1002/adhm.202505314)

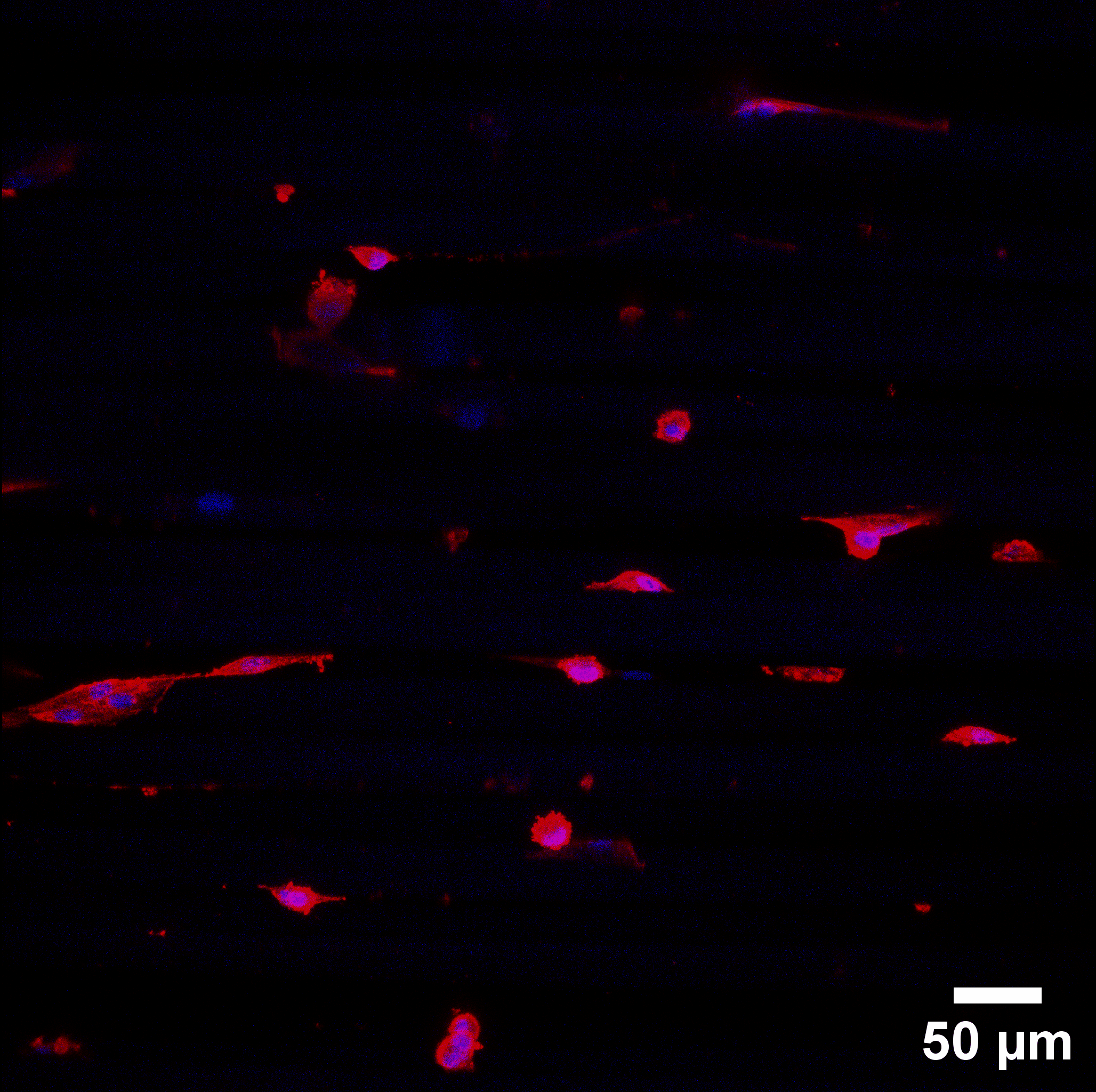

Supplement: Supplementary file 4 — Supporting File 4: adhm70862‐sup‐0004‐V3_Day1 PCL CASMCs. [file ADHM-15-0-s005.gif]

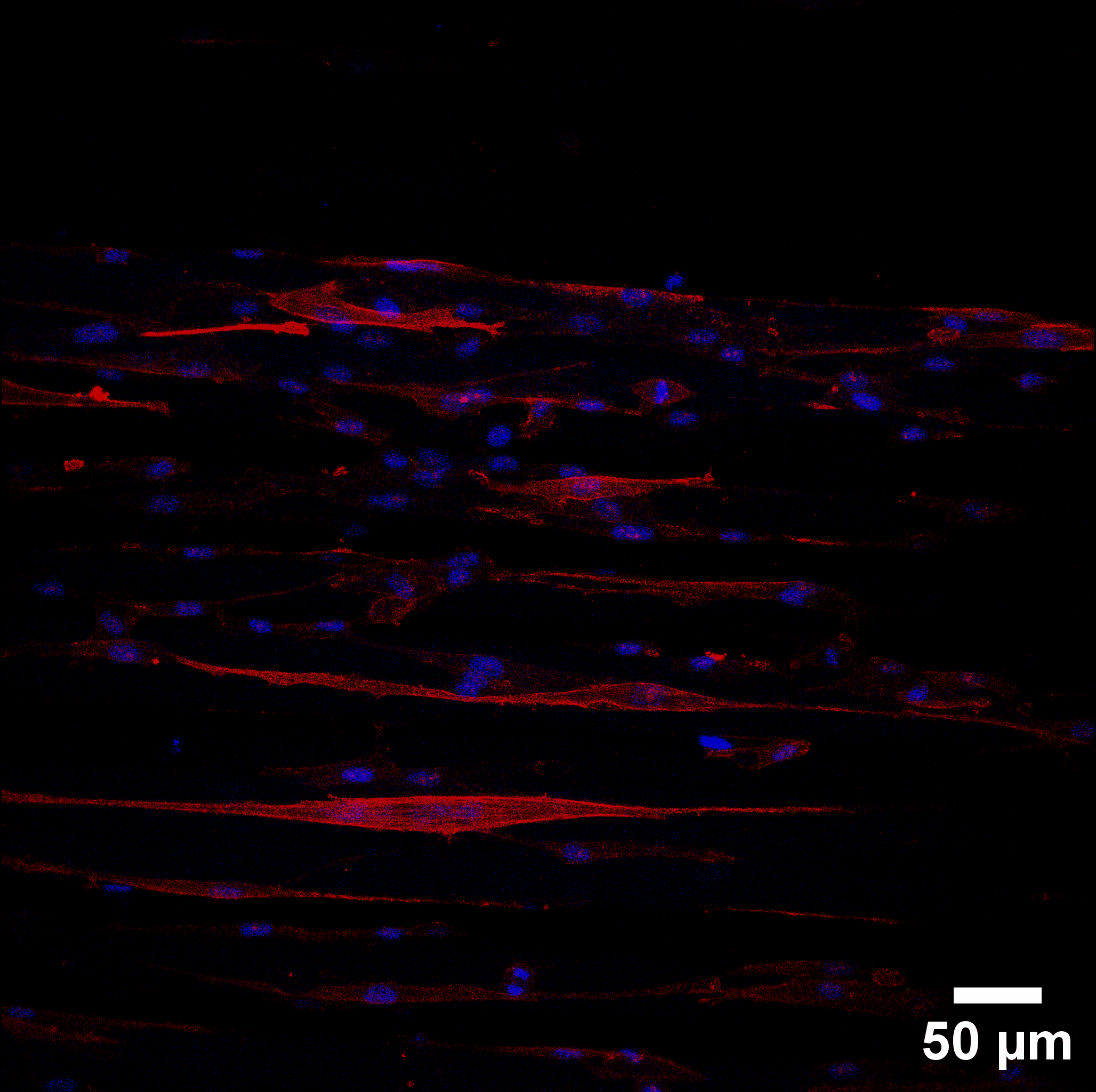

Supplement: Supplementary file 5 — Supporting File 5: adhm70862‐sup‐0005‐V4_Day1 HT CASMCs.gif. [file ADHM-15-0-s004.gif]

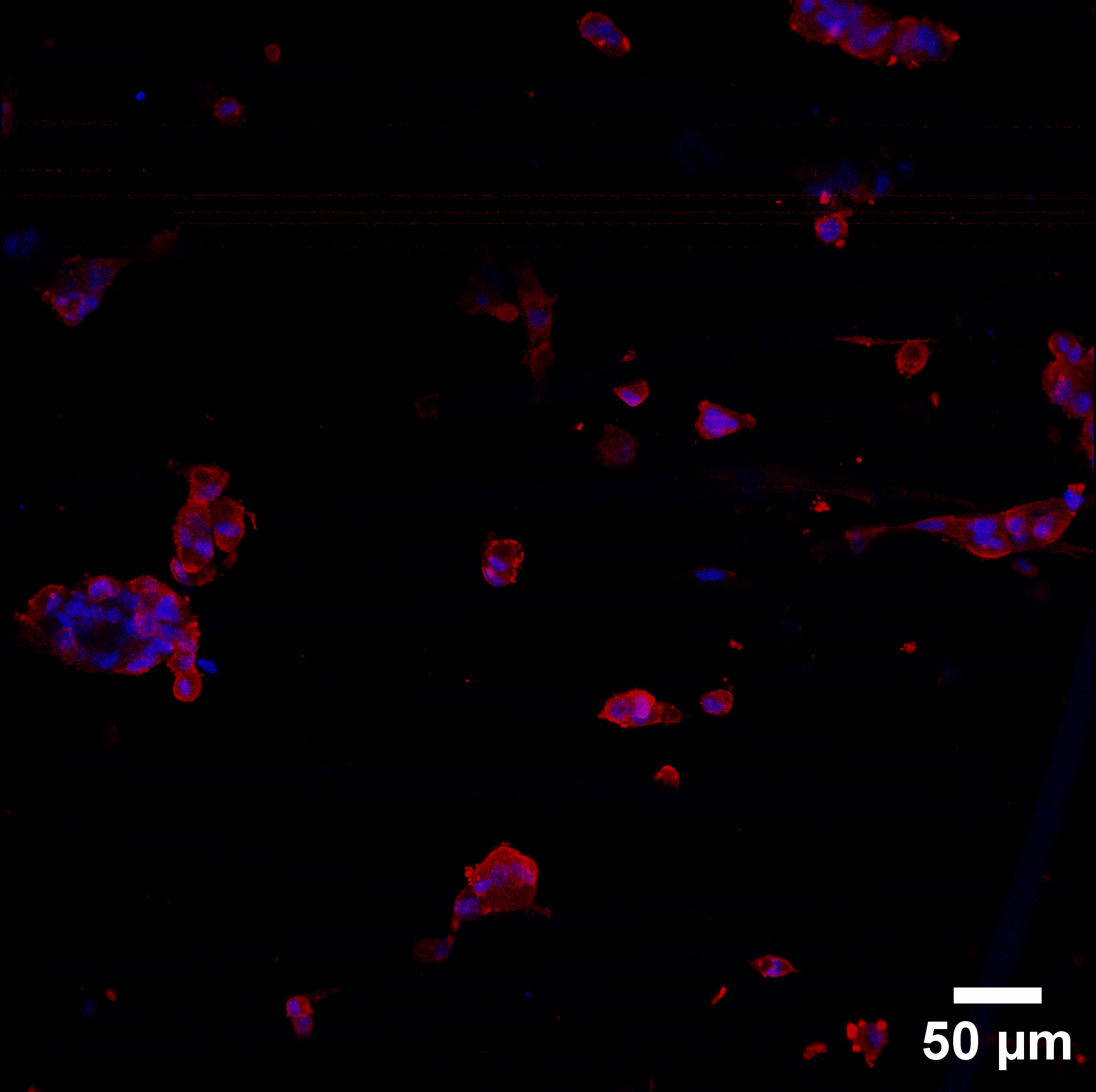

Supplement: Supplementary file 6 — Supporting File 6: adhm70862‐sup‐0006‐V5_Day 1 Hep CASMCs.gif. [file ADHM-15-0-s003.gif]
